# Anaerobic oxidation of propane coupled to nitrate reduction by a lineage within the class Symbiobacteriia

**DOI:** 10.1038/s41467-022-33872-y

**Published:** 2022-10-17

**Authors:** Mengxiong Wu, Jie Li, Andy O. Leu, Dirk V. Erler, Terra Stark, Gene W. Tyson, Zhiguo Yuan, Simon J. McIlroy, Jianhua Guo

**Affiliations:** 1 Australian Centre for Water and Environmental Biotechnology, Faculty of Engineering, Architecture and Information Technology, The University of Queensland, St Lucia, QLD Australia; 2 Centre for Microbiome Research, School of Biomedical Sciences, Queensland University of Technology (QUT), Translational Research Institute, Woolloongabba, QLD Australia; 3 Centre for Coastal Biogeochemistry Research, Faculty of Science and Engineering, Southern Cross University, Lismore, NSW Australia; 4 Metabolomics Australia (Queensland Node), Australian Institute for Bioengineering and Nanotechnology, The University of Queensland, St Lucia, QLD 4072 Australia

**Keywords:** Bacterial genomics, Environmental microbiology, Bacterial physiology

## Abstract

Anaerobic microorganisms are thought to play a critical role in regulating the flux of short-chain gaseous alkanes (SCGAs; including ethane, propane and butane) from terrestrial and aquatic ecosystems to the atmosphere. Sulfate has been confirmed to act as electron acceptor supporting microbial anaerobic oxidation of SCGAs, yet several other energetically more favourable acceptors co-exist with these gases in anaerobic environments. Here, we show that a bioreactor seeded with biomass from a wastewater treatment facility can perform anaerobic propane oxidation coupled to nitrate reduction to dinitrogen gas and ammonium. The bioreactor was operated for more than 1000 days, and we used ^13^C- and ^15^N-labelling experiments, metagenomic, metatranscriptomic, metaproteomic and metabolite analyses to characterize the microbial community and the metabolic processes. The data collectively suggest that a species representing a novel order within the bacterial class Symbiobacteriia is responsible for the observed nitrate-dependent propane oxidation. The closed genome of this organism, which we designate as ‘*Candidatus* Alkanivorans nitratireducens’, encodes pathways for oxidation of propane to CO_2_ via fumarate addition, and for nitrate reduction, with all the key genes expressed during nitrate-dependent propane oxidation. Our results suggest that nitrate is a relevant electron sink for SCGA oxidation in anaerobic environments, constituting a new microbially-mediated link between the carbon and nitrogen cycles.

## Abstract (web summary)

Anaerobic microorganisms can oxidize short-chain gaseous alkanes such as ethane, propane and butane using sulfate as electron acceptor. Here, the authors show that a bioreactor enrichment of a wastewater microbial community can perform anaerobic propane oxidation coupled to nitrate reduction.

## Introduction

A considerable amount of natural gas is generated from deep-sea sediments and hydrocarbon seeps in continental margins and terrestrial ecosystems^1,2^. Most of the natural gas released is consumed by microorganisms in anoxic zones before the gas diffuses into oxic environments and the atmosphere^3–5^. Studies of anaerobic oxidation of natural gas have focused on the potent greenhouse gas, methane, as the most abundant component (~60–90%)^6,7^. However, short chain gaseous alkanes (SCGAs), including ethane, propane, n-butane and *iso*-butane, are also substantial constituents of natural gas (up to ~20%)^8^, and are important precursors of ozone and organic aerosols^9^. The global atmospheric emissions of SCGAs are estimated to be 9.2–9.6 Tg yr^−1^ for ethane, 9.6–10.5 Tg yr^−1^ for propane, 10 Tg yr^−1^ for butane and 4.2 Tg yr^−1^ for *iso*-butane^10^.

Anaerobic oxidation of methane (AOM) has been extensively studied and is relatively well understood^7,11–17^. Anaerobic methanotrophic archaea (ANME) oxidize methane via the reverse methanogenesis pathway, including the key methyl-CoM reductase (MCR) complex. The ANME shuttle electrons from methane to syntrophic sulfate-reducing bacteria (SRB)^11,12^ or directly to the reduction of nitrate and metal oxides^13–16^. The bacterium *‘Candidatus* Methylomirabilis oxyfera*’* performs AOM via the “intra-aerobic” methane oxidation pathway using nitrite as the sole electron acceptor^17^. Microorganisms likely also couple the oxidation of SCGAs to the reduction of these electron acceptors under anoxic conditions^18^. *‘Ca*. M. oxyfera*’* were shown capable of oxidizing ethane and propane via the methane monooxygenase, although it is yet to be shown whether these carbon sources support growth^19^. To date, sulfate has been the sole electron sink identified to support anaerobic oxidation of SCGAs^20–25^. The deltaproteobacterial isolate *Desulfosarcina aeriophaga* BuS5 oxidizes propane and butane via the fumarate addition pathway generating alkyl-substituted succinates, a process mediated by alkylsuccinate synthase (ASS)^20,21^, and directly reduces sulfate to sulphide. An enrichment of the archaeon ‘*Candidatus* Syntrophoarchaeum’ was found to activate butane via butyl-coenzyme M formation, a process catalysed by a divergent MCR, with reducing equivalents channelled to syntrophic SRB partners^23^. Similarly, anaerobic ethane oxidation was linked to related ‘*Candidatus* Argoarchaeum ethanivorans’, which also encoded an MCR-like complex and was suggested to form a syntrophic relationship with SRB^22^. However, an archaeon able to mediate anaerobic propane oxidation directly coupled to sulfate reduction is yet to be identified.

Like sulfate, nitrate is a common electron acceptor in natural ecosystems^26^, and is used by ANME archaeon ‘*Ca*. Methanoperedens nitroreducens’ for AOM^13^. Nitrate reduction coupled to the oxidation of SCGAs (Eq. (1), using propane as an example) is also more thermodynamically feasible compared to the reactions of SCGA oxidation coupled to sulfate reduction (△G^o’^= −102 kJ/mol propane)^20^, but is yet to be linked to any microbial lineage.

Here we combined mass and electron balance tests, ^13^C- and ^15^N-labelling experiments, 16S rRNA gene amplicon sequencing, metabolite analyses and multi-omics approaches to provide evidence that a new bacterial species within the class Symbiobacteriia performs anaerobic oxidation of propane coupled to nitrate reduction.

## Results and discussion

### Enrichment of a microbial consortia able to couple anaerobic propane oxidation to nitrate reduction

**Fig. 1:**
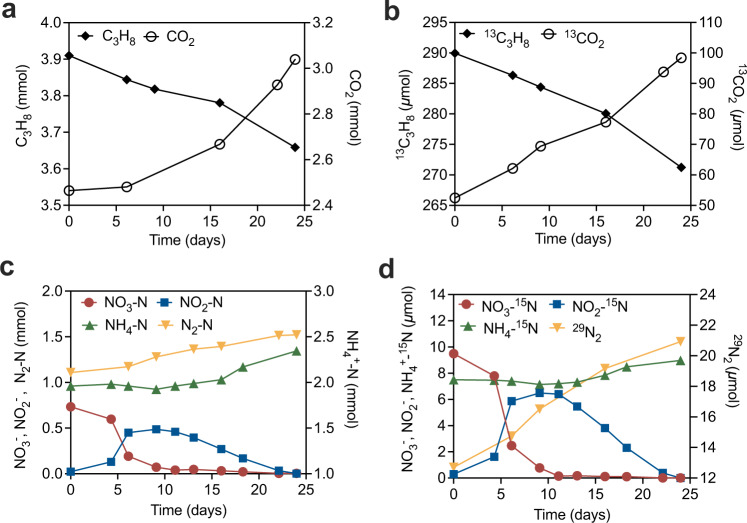
Anaerobic oxidation of propane coupled to nitrate reduction by the enrichment culture during the isotope labelling test. **a** Conversion of C_3_H_8_ to CO_2_, **b** ^13^C_3_H_8_ to ^13^CO_2_, **c** NO_3_^−^ to NH_4_^+^ and N_2_ with transitory formation of NO_2_^−^, **d** ^15^NO_3_^−^ to ^15^NH_4_^+^ and ^29^N_2_ with transitory formation of ^15^NO_2_^−^. Source data are provided as a Source Data file.

In this study, an anaerobic bioreactor seeded with biomass from a wastewater treatment facility was pulse-fed with propane and nitrate for more than 1000 days. Long-term performance data showed simultaneous consumption of propane and nitrate, with accumulation of ammonium, dinitrogen gas and transient accumulation of nitrite (Supplementary Fig. 1). No nitrate consumption was observed in the control incubations (without the addition of enrichment culture biomass or propane, Supplementary Fig. 2), suggesting nitrate reduction (to nitrite, ammonium and N_2_) coupled to propane consumption in the bioreactor was microbially mediated. To confirm nitrate-dependent anaerobic propane oxidation reaction, and to verify the final products thereof, biomass from the parent reactor was incubated in batch experiments with ^13^C-labelled propane (^13^CH_3_^13^CH_2_^13^CH_3_) and ^15^N-labelled nitrate (^15^NO_3_^−^) added to ~8% and ~1% of the total propane and total nitrate, respectively. The amount of total CO_2_ and ^13^C-labelled CO_2_ increased, accompanied by a decrease in C_3_H_8_ and ^13^C_3_H_8_ (Fig. 1a, b). The ratio (2.42) between the total ^13^CO_2_ produced (46 μmol) and total ^13^C_3_H_8_ consumed (19 μmol) was close to the theoretical stoichiometric ratio of 3:1. The carbon balance suggests that propane was oxidised with CO_2_ as the final product. The total NO_3_^−^ consumption (0.73 mmol) was consistent with the combined total N_2_ and NH_4_^+^ production (0.8 mmol) (Fig. 1c). Similarly, the ^15^NO_3_^−^ consumption (9.38 μmol) was close to the ^15^N in ^29^N_2_ (1.1–1.5% of total N_2_), ^30^N_2_ (0.03% of total N_2_), and ^15^NH_4_^+^ produced, which amounted to 9.71 μmol in combination (Fig. 1d). These results confirmed the reduction of nitrate to dinitrogen gas and ammonium. The electrons generated from anaerobic oxidation of propane (AOP, 5.03 mmol) were close to the electron demand for NO_3_^−^ reduction to N_2_ and NH_4_^+^ (5.15 mmol), suggesting electrons generated by AOP were used for denitrification and dissimilatory nitrate reduction to ammonia (DNRA), consistent with the following reactions^27^:

1 $${{{{{{{{\rm{C}}}}}}}}}_{3}{{{{{{{{\rm{H}}}}}}}}}_{8}+10{{{{{{{{\rm{NO}}}}}}}}}_{3}^{-}\to 10{{{{{{{{\rm{NO}}}}}}}}}_{2}^{-}+3{{{{{{{{\rm{CO}}}}}}}}}_{2}+4{{{{{{{{\rm{H}}}}}}}}}_{2}{{{{{{{\rm{O}}}}}}}},\,\varDelta {{{{{{{{\rm{G}}}}}}}}}^{^\circ {\prime} }=-1347.7\,{{{{{{{\rm{kJ}}}}}}}}/{{{{{{{\rm{mol}}}}}}}}\,{{{{{{{\rm{propane}}}}}}}}$$

2 $$3{{{{{{{{\rm{C}}}}}}}}}_{3}{{{{{{{{\rm{H}}}}}}}}}_{8}+10{{{{{{{{\rm{NO}}}}}}}}}_{2}^{-}+20{{{{{{{{\rm{H}}}}}}}}}^{+}\to 10{{{{{{{{\rm{NH}}}}}}}}}_{4}^{+}+9{{{{{{{{\rm{CO}}}}}}}}}_{2}+2{{{{{{{{\rm{H}}}}}}}}}_{2}{{{{{{{\rm{O}}}}}}}},\,\varDelta {{{{{{{{\rm{G}}}}}}}}}^{^\circ {\prime} }=-1192.3\,{{{{{{{\rm{kJ}}}}}}}}/{{{{{{{\rm{mol}}}}}}}}\,{{{{{{{\rm{propane}}}}}}}}$$

3 $$3{{{{{{{{\rm{C}}}}}}}}}_{3}{{{{{{{{\rm{H}}}}}}}}}_{8}+20{{{{{{{{\rm{NO}}}}}}}}}_{2}^{-}+20{{{{{{{{\rm{H}}}}}}}}}^{+}\to 10{{{{{{{{\rm{N}}}}}}}}}_{2}+9{{{{{{{{\rm{CO}}}}}}}}}_{2}+22{{{{{{{{\rm{H}}}}}}}}}_{2}{{{{{{{\rm{O}}}}}}}},\,\varDelta {{{{{{{{\rm{G}}}}}}}}}^{^\circ {\prime} }=-2384.8\,{{{{{{{\rm{kJ}}}}}}}}/{{{{{{{\rm{mol}}}}}}}}\,{{{{{{{\rm{propane}}}}}}}}$$

**Fig. 2:**
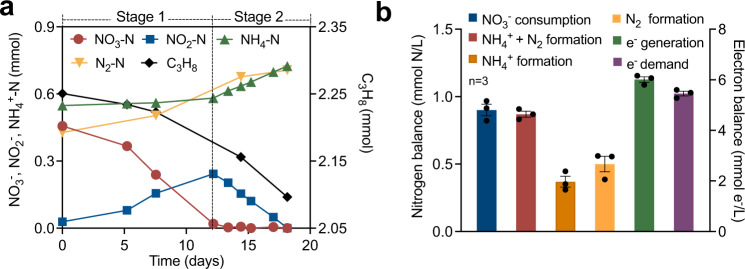
Mass balance and electron balance batch tests. **a** Typical biochemical profile of the systems (started on Day 1050) showing simultaneous nitrate and propane consumption with transitory formation of nitrite, and production of dinitrogen gas and ammonium. Nitrate reduction appeared to occur in two distinct phases. In Stage 1, nitrate was converted to nitrite and dinitrogen gas with negligible ammonium production; while in Stage 2, both dinitrogen gas and ammonium were generated from accumulated nitrite. **b** Average nitrogen- and electron balances were calculated from the three batch tests (for complete data and calculation see Supplementary Table 1). Error bars represent standard errors. Source data are provided as a Source Data file.

Stoichiometric experiments were conducted both in the parent reactor and in batch incubations seeded with biomass from the parent system, to more accurately establish the nitrogen and electron balances. The nitrate/nitrite reduction appeared to occur in two distinct stages (Fig. 2a). In Stage 1 (before nitrate depletion), nitrate was converted to nitrite and dinitrogen gas with negligible ammonium production. In Stage 2 (after nitrate depletion), both dinitrogen gas and ammonium were generated from nitrite accumulated in Stage 1. The observation that DNRA predominantly occurred when nitrate became limiting is consistent with previous studies^28–30^. The ratio between nitrate consumption to dinitrogen gas and ammonium production in triplicate batch tests (Fig. 2a, Supplementary Fig. 3) was 1.03 ± 0.05 (Fig. 2b, Supplementary Table 1), suggesting that nitrate was finally fully converted to dinitrogen gas (57.3 ± 5.5%) and ammonium (42.7 ± 5.5%). The ratio between electron production (calculated from propane oxidation to CO_2_) and electron consumption (nitrate reduction) was 1.10 ± 0.01 (Fig. 2b, Supplementary Table 1), suggesting nitrate reduction was the primary electron sink for propane oxidation.

### Characterization of the microbial community of the enrichment

**Fig. 3:**
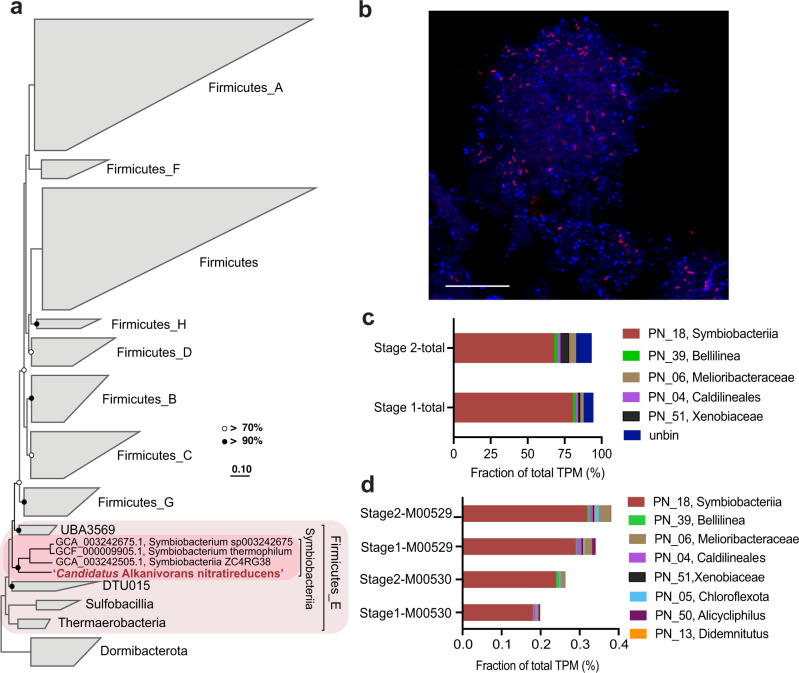
Phylogenetic affiliation and a fluorescent micrograph of ‘*Ca*. A. nitratireducens’, and relative gene expression profiles for the enrichment culture community. **a** Genome-based phylogenetic tree. The ‘*Ca*. A. nitratireducens’ genome from this study is highlighted in red text. Black and white dots represent >90% and >70% bootstrap values, respectively. The scale bars indicate amino acid substitutions per site. **b** A composite fluorescence micrograph of the enrichment culture hybridized with the SYMB-1018 FISH probe (Cy3, red; targeting *‘Ca*. A. nitratireducens*’*) and stained with DAPI (blue; all microbial cells). ‘*Ca*. A. nitratireducens’ cells appear magenta (blue + red). The scale bar indicates 20 μm. The representative image was selected based on the visual assessment of six separate hybridisation experiments. Consistent results were also obtained independently with two additional probes targeting the ‘*Ca*. A. nitratireducens’ population (Supplementary Fig. 8). **c**, **d** Relative expression of each genome and unbinned contigs, and microbial metabolism of interest for the genomes in the bioreactor. The total transcripts per million (TPM) was calculated for each gene. Genome sets with overall expression of total TPM > 1% are shown (**c**). KEGG annotation was used to identify ORFs coding for denitrification (M00529) and dissimilatory nitrate reduction to ammonium (M00530) pathways (**d**). Source data are provided as a Source Data file.

Community profiling with 16S rRNA gene amplicon sequencing revealed that a bacterial phylotype belonging to an unclassified lineage within the phylum Firmicutes was the most abundant population in the bioreactor (20.1% on Day 985, Supplementary Fig. 4), despite being undetectable in the inoculum. To obtain a representative genome for the dominant population, long (Nanopore) and short read (Illumina) metagenomic sequencing (Supplementary Data 1) was performed on biomass from the bioreactor sampled on Day 1040. Assembly and binning of the metagenomic data led to the recovery of 59 near complete genomes (≥70% completeness and ≤10% contamination based on CheckM, Supplementary Data 1, Supplementary Fig. 5) including 11 complete circularised genomes (Supplementary Data 1). The most abundant population was classified with the Genome Taxonomy Database (GTDB) to represent a novel order within the Class Symbiobacteriia of the Phylum Firmicutes (22.8% of relative abundance, Supplementary Table 2). We propose the name ‘*Candidatus* Alkanivorans nitratireducens’ for this bacterium based on its capability in nitrate-dependent propane degradation (see below). The 16S rRNA gene (1536 bp) recovered from the ‘*Ca*. A. nitratireducens’ MAG was identical to the abundant 16S rRNA gene amplicon sequence affiliated to the Firmicutes. The ‘*Ca*. A. nitratireducens’ MAG was complete and circularised with a size of 2.43 Mbp (Supplementary Fig. 6). A genome-based phylogenetic tree showed that the ‘*Ca*. A. nitratireducens’ was phylogenetically distinct from publicly available Symbiobacteriia (Fig. 3a). The average nucleotide identity (ANI) and amino acid identity (AAI) of the ‘*Ca*. A. nitratireducens’ genome, compared with those of its closest relatives in GTDB, i.e., Symbiobacteriia ZC4RG38, *Symbiobacterium* sp003242675 and *Symbiobacterium thermophilum* (<75.0% and 53.8–55.2% for ANI and AAI, respectively), supports the classification of ‘*Ca*. A. nitratireducens’ as a new order within the class Symbiobacteriia^31^. The 16S rRNA gene tree (Supplementary Fig. 7) also reveals that the ‘*Ca*. A. nitratireducens’ is phylogenetically distant from other classified Symbiobacteriia (<84.1% 16S rRNA gene identity)^31^ and has no close relatives in the Silva database. Three FISH probes (SYMB-1018, SYMB-624 and SYMB-186) were designed and individually applied to the bioreactor biomass to visualise the morphology and spatial arrangement of the ‘*Ca*. A. nitratireducens’ cells. Microscopic examination of the biomass revealed diffuse flocs with the cells appearing to be embedded in an extracellular polymeric substance-like matrix. ‘*Ca*. A. nitratireducens’ appeared as embedded rod shaped cells (~0.5 µm × 1.5 µm) that sometimes formed aggregates (up to ~50 µm) and were abundant and relatively evenly dispersed throughout the flocs (Fig. 3b and Supplementary Fig. 8). The morphology and spatial arrangement of the cells was consistent for all three probes, giving confidence in their specificity (Supplementary Fig. 8).

### Metabolic pathways of ‘*Ca*. A. nitratireducens’

**Fig. 4:**
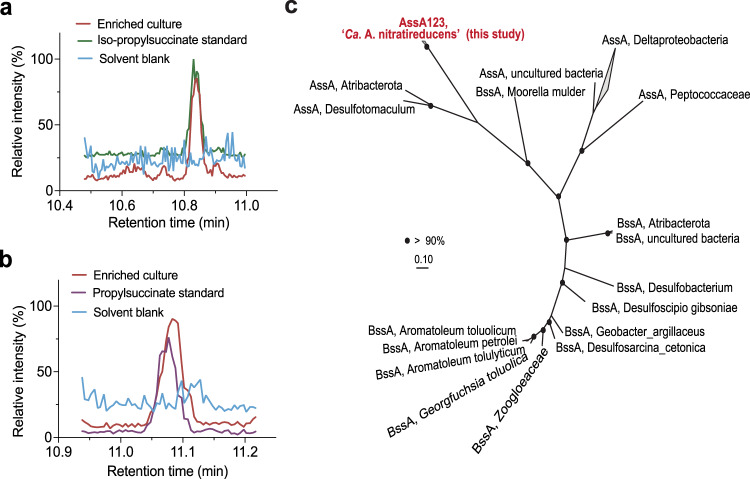
Detection of metabolic intermediate in propane oxidation and phylogenetic affiliation of AssA amino acid sequences in *‘Ca*. A. nitratireducens*’* genome. **a** Partial ion chromatograms (ion transition, *m/z*: 289 > 147.2) of the enriched culture extracts (*n* = 3, taken at various time points) revealed a peak at retention time 10.832 min, matching the peak of the *iso*-propylsuccinate standard. **b** A peak at retention time 11.078 min (ion transition, *m/z*: 289 > 147.1), corresponding to the peak from the propylsuccinate standard, was observed for cell extracts from the enriched culture (*n* = 3 at different sampling points). **c** Phylogenetic relationship of ‘*Ca*. A. nitratireducens*’* AssA (Red) to other AssA and BssA in the NCBI database. Three AssA subunits (AssA123) found in ‘*Ca*. A. nitratireducens*’* MAG formed a separate cluster. Bootstrap values >90% are shown as black dots on branch nodes. Scale bar represents amino acid substitutions per site.

**Fig. 5:**
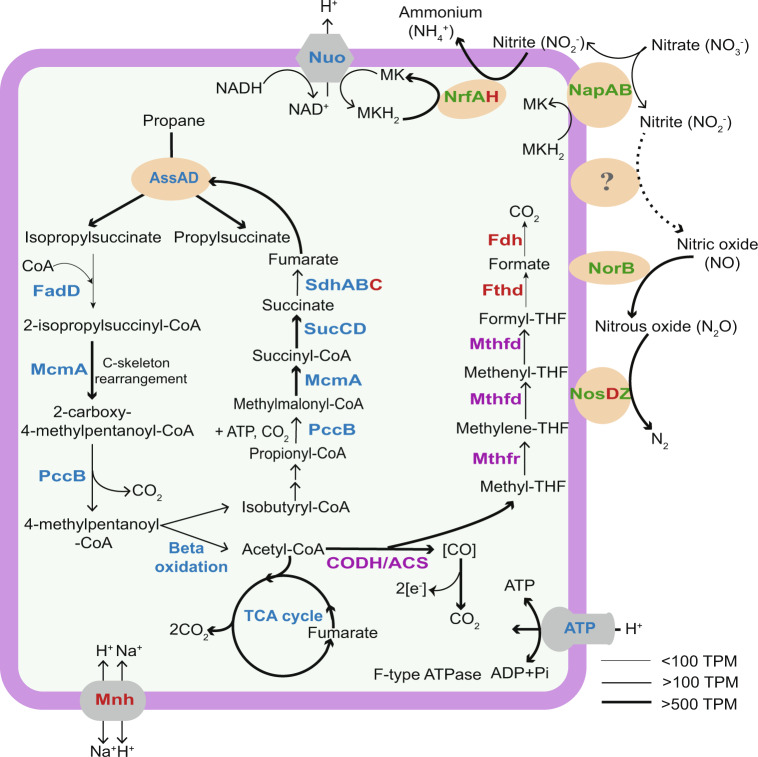
Proposed metabolic pathway for anaerobic propane oxidation coupled to nitrate reduction. The ‘*Ca*. A. nitratireducens’ enrichment culture utilized an alkylsuccinate synthase to activate propane to n- and *iso*-propylsuccinate. Acetyl-CoA is produced after carbon-skeleton rearrangement, decarboxylation and beta-oxidation. Acetyl-CoA oxidation to CO_2_ is catalysed by enzymes involved in the oxidative tricarboxylic acid cycle or reverse Wood–Ljungdahl pathway (purple labels). ‘*Ca*. A. nitratireducens’ harboured all the enzymes involved in denitrification (NapAB, NorB, NosZ, except NirS/K) and DNRA processes (NapAB, NrfAH) (green text). Normalized gene expression for ‘*Ca*. A. nitratireducens’ is indicated as TPM (total transcripts per million). Increasing line thickness shows increasing gene expression values. Enzymes labelled in blue, green, purple and bold text were fully or partially identified in the protein extracts, while red text indicates enzymes were not detected. Alkylsuccinate synthase, Ass; Long-chain acyl-CoA synthetase, FadD; Methylmalonyl-CoA mutase, Mcm; Propionyl-CoA carboxylase, Pcc; Succinate-CoA ligase, Suc; Succinate dehydrogenase, Sdh; Tricarboxylic acid, TCA; CO dehydrogenase:Acetyl-CoA synthase, CODH/ACS; Methylenetetrahydrofolate dehydrogenase, Mthfd; methylenetetrahydrofolate reductase, Mthfr; Formyltetrahydrofolate deformylase, Fthd; Formate dehydrogenase, Fdh; F-type H^+^-transporting ATPase, ATP; NADH-quinone oxidoreductase, Nuo; Multicomponent Na^+^ H^+^ antiporter, Mnh; Menaquinol, MKH_2_; Menaquinone, MK; Nitrate reductase, Nap; Cytochrome *c* 552 nitrite reductase, Nrf; Nitric oxide reductase, Nor; Nitrous oxide reductase, Nos.

Annotation of the ‘*Ca*. A. nitratireducens’ MAG identified genes for an ASS complex including two AssD (identical amino acid sequences) and three AssA subunits (89.3–90.7% similarities in amino acid sequences, Supplementary Fig. 9), which mediate the first step of anaerobic activation of SCGAs via fumarate addition^20,32^. Alignment of ‘*Ca*. A. nitratireducens’ AssA amino acid sequences with homologous sequences in the NCBI database confirmed the conservation of key amino acid residues (Gly828 and Cys489 in AssA1, Gly501 and Cys169 in AssA2, Gly828 and Cys489 in AssA3, Supplementary Fig. 10), which are important for the function of fumarate-adding enzymes^33^. Phylogenetic analyses revealed that the AssA and AssD of ‘*Ca*. A. nitratireducens’ are phylogenetically distant from fumarate addition enzymes (AssAD and BssAD) in the NCBI and UniRef databases (Fig. 4c; Supplementary Fig. 11). In addition, the ‘*Ca*. A. nitratireducens’ MAG harbours all genes required for the further degradation of propylsuccinate/*iso*-propylsuccinate, including methylmalonyl-CoA mutase genes (*mcmA*) for carbon-skeleton rearrangement, propionyl-CoA carboxylase genes (*pccB*) for decarboxylation and the key genes for beta-oxidation^34,35^ (Fig. 5; Supplementary Data 2). The acetyl-CoA generated from beta-oxidation may enter the oxidative tricarboxylic acid (TCA) cycle for complete oxidation to CO_2_ or for regeneration of fumarate for subsequent rounds of propane activation (Fig. 5). The isobutyryl-CoA could be oxidized, via methylmalonic semialdehyde, to propionyl-CoA^36^, which could be utilized to regenerate fumarate via the methylmalonyl-CoA pathway (Fig. 5). Genes for CO dehydrogenase:Acetyl-CoA synthase (CODH/ACS) and the reverse Wood–Ljungdahl (WL) pathway were identified in ‘*Ca*. A. nitratireducens’, suggesting that terminal oxidation of acetyl-CoA to CO_2_ can be mediated by CODH/ACS and stepwise dehydrogenation of the derived C1-units^37,38^ (Fig. 5; Supplementary Data 2; Supplementary Table 3). This WL pathway for CO_2_ production is consistent with that proposed for the sulfate-dependent propane degrading *Desulfosarcina aeriophaga* BuS5^21^. Currently, the Class of Symbiobacteriia only includes 3 genomes in the GTDB database and no metabolic models are available for anaerobic propane metabolisms. Therefore, further studies are required to construct metabolic models for ‘*Ca*. A. nitratireducens’ in order to calculate the carbon flows for TCA and WL pathways and to understand how the cells regulate these two pathways.

Metatranscriptomic and metaproteomic analyses revealed that genes involved in the proposed pathways for complete anaerobic propane oxidation to CO_2_ were highly expressed in ‘*Ca*. A. nitratireducens’, and their corresponding gene products were also detected in protein extracts after propane addition (with formyltetrahydrofolate deformylase and formate dehydrogenase as exceptions, Fig. 5; Supplementary Data 2), indicating ‘*Ca*. A. nitratireducens’ played an active role in AOP during both Stage 1 and 2. A search of the entire metagenome library suggested no other populations in the system encoded known genes linked to AOP, including genes for ASS^20,32^ and alkyl-coenzyme M reductase^22,23^. The relative activity of co-existing microbial populations (collectively accounting for 14–22% of total transcriptome reads) was also substantially lower than that of *‘Ca*. A. nitratireducens’ (64–83% of total transcriptome reads, Fig. 3c). These results strongly suggest that *‘Ca*. A. nitratireducens’ was responsible for AOP in the system.

Cell extracts from the enrichment biomass were analyzed for key metabolites of the activation pathway using ultra-high-sensitivity triple quadrupole mass spectrometry. Total ion chromatograms indicated identical retention time for the propylsuccinate standards and extracted metabolites (Supplementary Fig. 12). Mass peaks corresponding to *iso*-propylsuccinate (*m/z*: 289 > 147.2) and propylsuccinate (*m/z*: 289 > 147.1) standards were detected in the cell extracts (Fig. 4a, b). These findings support the hypothesis that ‘*Ca*. A. nitratireducens’ activates propane through homolytic C-H bond cleavage at both primary and secondary carbon atoms, and with addition of fumarate, yields propylsuccinate and *iso*-propylsuccinate. In addition, *iso*-propylsuccinate (5.96 nM) was more abundant than propylsuccinate (2.33 nM) in the culture extracts, suggesting the secondary carbon atom activation generating *iso*-propylsuccinate is the main route of propane oxidation (Fig. 5).

The ‘*Ca*. A. nitratireducens’ MAG also contains all the genes required for DNRA (Fig. 5; Supplementary Table 4), including a nitrate reductase (*napAB*) which catalyses the reduction of nitrate to nitrite, and cytochrome *c* nitrite reductases (*nrfAH*) responsible for reducing generated nitrite to ammonium. Metatranscriptomic results revealed that *napAB* genes encoded by ‘*Ca*. A. nitratireducens’ were more highly expressed in Stage 1 relative to Stage 2, consistent with the higher concentration of nitrate in Stage 1 (Supplementary Fig. 13a). In addition, ‘*Ca*. A. nitratireducens’ *nrfAH* was highly expressed in Stage 2 where the bulk of ammonium was generated (Supplementary Fig. 13b). Moreover, the catalytic subunits of nitrate reductase (NapAB) and cytochrome *c* nitrite reductase (NrfA) were also identified in protein extracts (Fig. 5; Supplementary Table 4). Other members of the community also expressed genes for dissimilatory nitrate reduction pathways (denitrification and DNRA; Fig. 3d), but at substantially lower levels compared to *‘Ca*. A. nitratireducens’.

The contribution of ‘*Ca*. A. nitratireducens’ to the observed production of dinitrogen gas is not completely clear given the closed representative MAG lacks an identifiable nitric oxide-producing nitrite reductase (*nirS/K*). The MAG has the annotated potential for the last two steps of denitrification; encoding nitric oxide reductase (*norB*) and nitrous oxide reductase (*nosZD*) for the conversion of nitric oxide to dinitrogen gas (Fig. 5; Supplementary Table 4). These genes were highly expressed in both stages and were detected in the protein extracts (Supplementary Table 4), indicating that this microorganism is contributing to the reduction of nitric oxide to dinitrogen gas. These observations suggest that ‘*Ca*. A. nitratireducens’ may utilise a novel gene or novel pathway for nitrite reduction to nitric oxide. Indeed, several other microbes, such as *Bacillus vireti* and methylotrophic bacteria, have been reported to produce nitric oxide even though they do not harbour *nirS/K* genes^39–41^. It is also possible that other members of the community contribute to the observed denitrification to dinitrogen gas, or the nitrite reduction to nitric oxide. However, the expression of *nirS/K* genes and other denitrification genes was relatively low for all other populations (Fig. 3d), suggesting that ‘*Ca*. A. nitratireducens’ was responsible for the bulk of both the nitrogen- and carbon transformations in the system.

The identification of an anaerobic propane-oxidizing firmicute expands the known phylogenetic diversity of microbial lineages which mediate anaerobic oxidation of SCGAs in the environment, being previously shown for members of the Deltaproteobacteria and archaeal phylum Halobacteriota. To our knowledge, this study is the first to identify a microorganism mediating nitrate-dependent anaerobic propane oxidation (n-DAPO). Given the prevalence of nitrate in various natural and engineered ecosystems, combined with increasing atmospheric emissions of propane due to oil and natural gas production^42^, the previously undescribed n-DAPO process likely occurs in natural environments, representing an overlooked link between global carbon and nitrogen cycling. The newly discovered n-DAPO process may play an important role in reducing the negative impact of propane on air quality and on climate, which warrants further investigations. Although propane is a less potent greenhouse gas compared to methane, it can react with the hydroxyl radical, resulting in increased production of ozone^9,43^. Also, propane contributes to the formation of NO_2_ and peroxyacetyl nitrate, two significant air pollutants^43^. The identification and characterisation of ‘*Ca*. A. nitratireducens’ contributes to a better understanding of the role of microorganisms in regulating SCGA emissions.

## Methods

### Description of new species

#### Etymology

Alkanivorans (al.ka.ni.vo’rans. N.L. neut. n. alkanum, alkane; L. pres. part. vorans, devouring; N.L. masc. n. Alkanivorans, an alkane eater). nitratireducens (ni.tra.ti.re.du’cens. N.L. masc. n. nitras (gen. nitratis), nitrate; L. pres. part. reducens, converting to a different state; N.L. part. adj. nitratireducens, reducing nitrate). This name implies an organism capable of consuming propane and reducing nitrogen-related compounds.

#### Locality

Enriched from a mixture of anaerobic digestion sludge and activated sludge from a wastewater treatment plant in Brisbane, Australia.

#### Diagnosis

Anaerobic, propane-oxidizing, nitrate-reducing bacteria, typically observed as bacillus shaped cells approximately 0.5 (diameter) × 1.5 µm (length), sometimes forming clusters. Mesophilic in terms of temperature and pH (enriched at 22–25 °C and pH 7–8).

### Enrichment bioreactor operation

Without an easy access to sediments in a propane-rich environment, such as deep-sea gas seeps or hot spring sediments, a mixture of approximately 100 mL of anaerobic digestion sludge and 50 mL of activated sludge from a full-scale wastewater treatment plant (Brisbane, Australia) was used as inoculum for the bioreactor enrichment. Wastewater typically contains a wide range of organic substrates enabling the growth of a diverse range of microorganisms. Also, small quantities of propane could also be generated in anaerobic digestion systems^44^. We hypothesised that some propane oxidizing microorganisms could be present in such systems. The inoculum was incubated in a 2.3 L bioreactor with 1.84 L anoxic synthetic medium prepared as described previously^45^, leaving a headspace of 0.46 L. The propane partial pressure in the headspace was maintained between 0.9 and 1.4 atm by periodically flushed the liquid and headspace with pure propane gas (99.99%, Coregas, Australia). Helium was manually injected into the headspace to pressurize the reactor up to 1.5 atm. Nitrate was pulse-fed to the reactor by manual injection of concentrated stock solution (80 g NO_3_^−^-N l^−1^). The bioreactor was mixed continuously with a magnetic stirrer (IKA, Labtek, Australia) at 400 rpm and incubated at room temperature (22 ± 2 °C). The pH was controlled between 7 and 7.5 by manually adding 1 M anoxic HCl solution. Every 1–3 months, 200 mL of the supernatant was exchanged with fresh synthetic medium. A liquid sample (one mL) was withdrawn 2–3 times per week via the bioreactor sampling port, and 0.22 μm filtered (polyethersulfone filter, Millex, USA) for the measurement of nitrate, nitrite and ammonium. A gas sample (100 μL) was taken from the headspace using a gas-tight syringe (Hamilton, USA) 2–3 times per week for the measurement of propane and dinitrogen gas.

### Batch tests for stoichiometric determination

For stoichiometric determination of nitrate reduction and anaerobic propane oxidation, propane and nitrogen concentrations in the headspace of the 2.3 L bioreactor were periodically measured in addition to nitrate, nitrite and ammonium from Day 990 to 1000. In addition, two batch tests were also conducted in 650 mL glass vessels with a subsample of 500 mL biomass anaerobically transferred from the 2.3 L bioreactor (Supplementary Table 5). The biomass was then flushed with argon gas (99.99%, Coregas, Australia) for 20 min to remove dissolved propane in the liquid. Approximately 45 mL propane gas was added to the headspace as the sole electron donor. Enriched cultures without propane, and sterile synthetic medium with propane were set up as controls (Supplementary Table 5). Each batch test was conducted in triplicate.

### Isotope labelling batch tests

For the isotope labelling experiments, a subsample of 500 mL biomass from the bioreactor was transferred anaerobically to a 650 mL glass vessel. The biomass was flushed with pure propane gas (99.99%, Coregas, Australia) for 20 min. Approximately 12 mL ^13^C-labelled propane (^13^CH_3_^13^CH_2_^13^CH_3_, 99 atom % ^13^C, Sigma) was injected into the headspace through the septum. Approximately 1 mL nitrate stock solution (10 g N L^−1^) containing ~1% ^15^N-labelled sodium nitrate (98 atom % ^15^N, Sigma) was added to achieve a concentration of ~20 mg N L^−1^. Using a gas-tight syringe, gas samples were taken periodically from the headspace (Hamilton, USA) and then injected into helium-flushed vials (Exetainer, UK). Liquid samples were collected, filtered (0.22 μm), and stored at −20 °C until analysis for ^15^NO_3_^−^, ^15^NO_2_^−^ and ^15^NH_4_^+^. To quantify the CO_2_ produced in the liquid phase, unfiltered samples were injected into Exetainer vials, acidified with 1 M HCl solution, and equilibrated with the headspace for at least 0.5 h before CO_2_ determination.

### Chemical analysis

Nitrate, nitrite and ammonium concentrations in filtered samples were determined using a Lachat QuickChem8000 flow injection analyzer (Lachat Instrument, Milwaukee, WI). Propane, nitrogen and carbon dioxide concentrations in the headspace were quantified with a gas chromatograph (GC, 7890 A, Agilent, USA) equipped with a thermal conductivity detector and a Shincarbon ST packed column (2 m × 2.0 mm). The gas chromatograph was operated using argon as the carrier gas (flow rate, 28 mL min^−1^). The oven, injector and detector temperatures were maintained at 220, 250 and 260 °C, respectively. Propane, nitrogen and carbon dioxide concentrations were calculated based on an external standard curve.

Isotopically labelled samples containing nitrate and nitrite were divided into two equal subsamples. For one subsample, nitrate and nitrite ^15^N fractions were measured with a Thermo Delta V isotope ratio mass spectrometer (IRMS) after conversion to N_2_O^46^. For the other subsample, nitrite was removed using 4% (wt/vol) sulfamic acid in 10% HCl^47^, and the ^15^N fraction in the remaining nitrate was measured by IRMS. The fraction of ^15^N in nitrite was then calculated using the equation: ^15^N fractions in (NO_3_^−^ + NO_2_^−^) × total amount of (NO_3_^−^ + NO_2_^−^) = ^15^N fraction in NO_3_^−^ × total amount of NO_3_^−^ + ^15^N fraction in NO_2_^−^ × total amount of NO_2_^−^. Using a microdiffusion method^48,49^, ammonium was trapped in GF/D filters (Whatman, UK) which were then combusted for isotopic analysis using IRMS. ^29^N_2_, ^30^N_2_, ^13^C-labelled propane and ^13^CO_2_ in gas samples were determined with a gas chromatograph (7890 A, Agilent, Unite States) coupled to a quadrupole mass spectrometer (5957 C inert MSD, Agilent, Unite States). The gas chromatograph was equipped with a J&W HP-PLOT Q PT column (30 m × 530 μm) and was operated using helium as a carrier gas (flow rate 5.58 mL/min). The oven was maintained at 45 °C for 2 min, and then heated at a rate of 10 °C/min to 60 °C where it was held for 6 min. N_2_, CO_2_ and C_3_H_8_ were detected at 70 eV electron impact (EI) using the standard autotune procedure for mass calibration. Acquisition was performed in Total Ion Chromatography (TIC) for identification and in Selected Ion Monitoring (SIM) for monitoring m/z signals at 28, 29 and 30 Da (N_2_), 44 and 45 Da (CO_2_), 44 and 47 Da (C_3_H_8_) with a dwell time of 100 ms for each signal. Data processing was performed using the Chemstation program (Agilent, United States).

### 16 S rRNA gene amplicon sequencing

Biomass samples (10 mL) were taken from the enrichment bioreactor every 2–3 months and pelleted by centrifugation (8000 × *g* for 10 min) for DNA extraction. DNA was extracted using the FastDNA SPIN for Soil kit (MP Biomedicals, USA) according to the manufacturer’s protocol. DNA concentrations were measured using a Nanodrop spectrophotometer (Thermo Fisher Scientific, Wilmington, DE). Amplicon sequencing for the 16 S rRNA genes (V6 to V8 regions) was performed using the universal primer set 926 F (5ʹ-AAACTYAAAKGAATTGACGG-3ʹ) and 1392 R (5ʹ-ACGGGCGGTGTGTRC-3ʹ)^50^ on an Illumina MiSeq platform (Illumina, USA) at the Australian Centre for Ecogenomics (ACE; Brisbane, Australia). Sequencing results were processed using QIIME2^50^.

### Metagenomic sequencing and quality control of short- and long-reads

DNA extracted on Day 1040 (for the initial metagenome) and Day 1100 (for the shallow metagenome) was sequenced via short-read paired-end libraries using an Illumina Nextera XT DNA library preparation kit and the NextSeq500 (Illumina, USA) platform at ACE, based on the manufacturer’s protocol. Samples for shallow metagenomic sequencing were also taken at the same time as samples for metatranscriptomics sequencing to ensure the microbial community was consistent with the initial metagenome (Supplementary Fig. 14). The libraries generated 149 million and 1 million reads on average for the initial and shallow metagenome, respectively (Supplementary Data 1). Duplicates, adapters and bad quality bases in generated reads were removed using ReadTrim (https://github.com/jlli6t/ReadTrim) with parameter “–remove_dups–minlen 100” which internally calls FastQC (https://www.bioinformatics.babraham.ac.uk/projects/fastqc/), FastUniq-1.1^51^, cutadapt 2.10 and Trimmomatic-0.36^52^.

Biomass from the batch reactor (Day 1120) was also subjected to Nanopore long-read sequencing. DNA was extracted using the Qiagen PowerSoil Pro kit (Qiagen, Germany) and quality-checked using a combination of Qubit 1x dsDNA HS Assay Kit on the Qubit Flex Fluorometer (Thermo Fisher Scientific, Wilmington, DE) and the QIAxcel DNA High Resolution Kit on the QIAxcel Advanced system (Qiagen, Germany). Library preparation was completed according to the manufacturer’s protocol and sequenced on a PromethION (Oxford Nanopore Technologies, USA). Base-calling was performed using Albacore (https://github.com/Albacore/albacore), resulting in 73 million reads with quality > Q5. Among them, 12 million reads were longer than 1000 bp with read N50 of 6,135 bp. Adapters were trimmed using Porechop v0.2.4 (https://github.com/rrwick/Porechop).

### Recovery and evaluation of abundant genomes

Aviary (https://github.com/rhysnewell/aviary) was used for hybrid assembly and binning of short- and long-reads, internally calling several different assemblers and binning tools. Results were manually checked using Bandage^53^. Resulting bins were organized using DASTools 1.1.2^54^. Recovered genomes were further dereplicated using ‘dereplicate’ workflow in drep-3.2.2^55^ with default settings, resulting in a non-redundant genome set consisting of 59 high-quality genomes. Coverage of genome information and other details were viewed and manually checked using IGV 2.11.1^56^. Completeness and contamination of genomes were checked using CheckM v1.1.3^57^. Quality-trimmed short-reads were mapped to the final MAG set and unbinned contigs using bowtie 2.3.4.3. Mappings with an aligned length ratio over a read <75% or identity of aligned region <97% were removed. Abundance of each MAG was profiled using CoverM 0.6.1 (https://github.com/wwood/CoverM) with only quality primary mappings. NGA50 and other genome characteristics were calculated using Python package BioSut (https://github.com/jlli6t/BioSut).

### Functional annotation

For all MAGs and unbinned contigs, open reading frames (ORFs) were called and primary annotation of genomes was conducted using Prokka 1.14.5^58^ with the domain inferred from GTDB-Tk classification. The KEGG Orthology HMM database (accessed July 2021) was searched against using kofamscan 1.3.0^59^, selecting the top hit for each gene with e-value <1e-10 and maximal F-measure. UniRef100^60^ (accessed March 2020) was indexed with the NCBI taxonomy database and searched against using diamond^61^ v2.0.11.149 with ‘blastp–sensitive’. The top hit with e-value <1e-5 and identity > 30 was selected and mapped to the KEGG Orthology database. The eggNOG v5 database was searched against using emapper 2.1.5 in diamond mode. Conserved motif(s) present within the predicted genes related to propane oxidation and nitrogen metabolism were further verified using NCBI’s conserved domain search^62^. Annotated KO numbers were used for inferring the pathway encoded in each genome. Pathways were identified as ‘not expressed’ if missed blocks > 75% when total blocks > 5, or missed blocks > 1 when total blocks ≤ 5.

### Genome tree construction and classification

A bacterial genome tree was constructed using the Genome Taxonomy Database (GTDB r202) and recovered bacterial genomes with a concatenated set of 120 bacterial-specific conserved marker genes inferred from genomes. Briefly, marker genes in genomes were identified using Prodigal 2.6^63^ and aligned using HMMER 3.3^64^. Trees were inferred using FastTree 2.1.11^65^ with WAG + GMMA models. Bootstrapping was performed using GenomeTreeTk v0.1.6 (https://github.com/dparks1134/GenomeTreeTk) with 100 times nonparametric bootstrapping. Trees were visualized using ARB 6.0.6^66^ and imported into Adobe Illustrator (Adobe, USA) for further refinement. Classification of genomes was determined using ‘classify_wf’ workflow in GTDB-Tk v1.5.1^67^.

### Phylogenetic analyses of the 16S rRNA gene and alkylsuccinate synthase in ‘*Ca*. A. nitratireducens’

The 16S rRNA genes were identified in the ‘*Ca*. A. nitratireducens’ MAG and publicly available Symbiobacteriia genomes in database. These genes were compared against the SILVA 138 SSU database. Selected sequences and predicted 16S rRNA gene sequences were dereplicated using cd-hit 4.8.1^68^. In total, 112 16S rRNA sequences were collected and aligned using SSU-align v0.1.1^69^. The phylogenetic tree was inferred using FastTree 2.1.11^65^ with ‘-gtr -gamma’ parameters.

For phylogenetic analyses of AssA in ‘*Ca*. A. nitratireducens’, 24 reference AssA and BssA protein sequences, longer than 700 amino acids (accession numbers of reference sequences are provided in Supplementary Table 6), were downloaded from public databases and aligned using muscle 3.8.31^70^ with parameter ‘-diags1 -maxiters 5’. Gaps in msa were trimmed using trimAI 1.4.1^71^. The phylogenetic tree was inferred using FastTree 2.1.11^65^. For construction of both 16S rRNA gene and AssA amino acid trees, bootstrap value calculation and tree visualization were as per the genome tree construction.

### Metatranscriptomic sequencing and data analysis

Triplicate batch tests were conducted on Day 1100 in three 650 mL glass vessels with a subsample of 500 mL biomass in each vessel. The nitrate reduction in bioreactors was divided into two stages. In Stage 1, nitrate was mainly converted to nitrite and dinitrogen gas with negligible ammonium production, while in Stage 2 ammonium and dinitrogen gas were both produced. For the co-extraction of total RNA and DNA, 10 mL of an active enriched culture was collected from each batch test in each stage and preserved by adding RNAlater solution (Sigma-Aldrich) and left to stand at room temperature for 1 h. The mixture was then filtered through a sterilized cellulose nitrate filter (0.20 μm; Sartorius; Göttingen, Germany). To capture microorganisms that may pass through the 0.2 μm filter, the filtrate was further concentrated using an Amicon® Ultra Filter (Sigma-Aldrich) with molecular weight cut-off of 100 K. Total RNA and DNA in the concentrated filtrate and cellulose nitrate filter were then extracted by using the RNeasy Powersoil Total RNA kit with the RNeasy PowerSoil DNA Elution Kit (Qiagen, Germany) according to the manufacturer’s protocols. Trace genomic DNA was removed from the RNA extracts with a Turbo DNA-free kit (Thermo Fisher Scientific, USA) followed by a RNA Clean & Concentrator-5 Kit (Zymo Research, USA). The TruSeq Total RNA Library Prep with Ribo-Zero Plus kit was used for RNA library preparation following the manufacture’s protocol. The library was sequenced on a NextSeq500 (Illumina, USA) platform at ACE (Brisbane, Australia) in 2 × 75 cycles paired-end runs (Table S1).

Generated reads were trimmed using ReadTrim (https://github.com/jlli6t/ReadTrim) with parameter “–remove_adap –minlen 60”. An rRNA database was constructed from archaeal and bacterial 5 S rRNA from 5SRNAdb^72^, SSU database from SILVA^73^ v138, and LSU database from SILVA v132. Ribosomal RNA-like reads were removed using SortMeRNA 4.2.0^74^ with default settings and database constructed as above. Quality-trimmed reads were mapped to a dereplicated MAGs set and unbinned scaffolds using bowtie2. Alignments with aligned length <95% of read length or identity <97% were removed. Potential DNA contamination of RNA libraries were identified using RNAdir (https://github.com/jlli6t/RNAdir), a modified version of dirseq (https://github.com/wwood/dirseq). Following, a binomial test was conducted using scipy.stats.binomtest function. Mappings were used for calculation of TPM (total transcripts per million)^75^. Total expression levels of each genome were calculated as the sum of TPM of all coding sequences within each genome.

### Protein extraction and metaproteomics

For protein extraction, 10 mL of enrichment culture collected from transcriptomics batch tests was pelleted by centrifugation (18,000 × *g*, 4 °C), washed with 1 × PBS and stored at −80 °C until analysis. The cell pellets were lysed in 5% sodium dodecyl sulfate (SDS), incubated with 20 mM dithiothreitol (final concentration) at 70 °C for 60 min and then cooled to room temperature, followed by alkylation with 40 mM iodoacetamide (final concentration) in the dark for 30 min. Afterwards, 1.2% phosphoric acid (final concentration) and six volumes of S-Trap binding buffer (90% methanol, 100 mM final concentration of ammonium bicarbonate, pH 7.1) were added. Total protein was digested in a S-Trap Micro Spin Column (ProtiFi, Huntington, USA) according to the manufacturer’s protocol^76^. Briefly, the protein solution was loaded on the S-Trap filter and centrifuged at 4,000 g until all solution passed through. The filter was washed with 150 μL S-Trap binding buffer three times and digested with 1 μg sequencing-grade trypsin at 47 °C for 1 h. The digested peptides were sequentially eluted using 40 µl of 50 mM ammonium bicarbonate, 0.1% aqueous formic acid, 50% acetonitrile and 0.1% formic acid in H_2_O. The peptide solutions were dried before being resuspended in 20 *µ*l of 5% acetonitrile in H_2_O. The peptides were analysed by Liquid Chromatography-tandem Mass Spectrometry (LC-MS/MS) using a Dionex Ultimate 3000 RSLCnano-LC system coupled to a Q-Exactive^TM^ H-X Hybrid Quadrupole-Orbitrap™ mass spectrometer (Thermo Scientific^TM^). Raw sequencing data were processed by searching against the annotated metagenomes of ‘*Ca*. A. nitratireducens’ in Thermo Proteome Discoverer (version 2.2.0.388). The identified proteins contained at least 1 unique peptide with a stringency cut-off of false discovery rate (FDR, *q* value) less than 0.05.

### Metabolite extraction

For preparation of the metabolite extract, 5 mL of enrichment culture was collected from the bioreactor. The cells were harvested by centrifugation (8000 × *g*, 10 min, 4 °C) and then resuspended in 1 mL acetonitrile-methanol-water (4:4:2, vol/vol/vol) mixture in lysing matrix tubes (MP Biomedicals) with glass beads. The cells were lysed using a bead-based homogenizer operated for 5 cycles of reciprocal shaking at low speed (3000 rpm, 50 s) with cooling on ice (15 s) between the homogenization steps. The extracts were separated from cell debris and glass beads by centrifugation at 18,000 g for 10 min at 4 °C and stored at −80 °C until analysis.

### Mass spectrometry of standards and cell extracts

The custom synthesized standards (propyl and isopropyl succinate, Best of Chemicals, USA) and cell extracts were dried in a rotational vacuum concentrator (Concentrator Plus, Eppendorf), and then derivatized in 20 μL methoxyamine chloride (30 mg/ml in pyridine) with continuous mixing for 2 h at 37 °C. *N*,*O*-bis (trimethylsilyl) trifluoroacetamide (BSTFA, 20 μL) containing 1% trimethylchlorosilane (TMCS) was then added. Samples were incubated with continuous shaking for 30 min at 37 °C and then analysed using an ultra-high-sensitivity triple quadrupole GC/MS-TQ8050 system (Shimadzu, Japan). The GC system was equipped with an EI ionisation source and operated in multiple reaction monitoring (MRM) mode for the detection of compounds. Helium was used as a carrier gas at a constant flow rate of 1.0 mL/min. One microliter of the derivatised sample was injected into a PTV injector in split mode 1:10 with an injection temperature of 280 °C. Separations were carried out in a DB-5ms column (95% polydimethylsiloxane, 30 m × 0.25 mm × 1 μm) (Agilent JW Scientific). The oven temperature was initially held for 1 min at 120 °C, then increased to 220 °C at a rate of 8 °C/min, and finally increased to 320 °C at 50 °C/min and held for 1 min. The ion source temperature was set at 200 °C. The specific mass spectrometric parameters were tailored for each compound to monitor the fragmentation ions for each analyte (See Supplementary Table 7). Two fragment ions from the EI ion source were nominated and then further fragmented in the collision cell. The three most abundant transients were chosen to monitor. Transient 1 was used as quantifier and the other two as qualifiers.

### Fluorescence in situ hybridization (FISH)

FISH was performed essentially as detailed by Daims, et al.^77^. Biomass from the bioreactor was fixed with 4% paraformaldehyde (w/v) and stored in 50% ethanol in PBS at −20 °C. FISH probes were designed using ARB software^70^ against the Silva SSU Ref NR99 database release 138^73^ including relevant sequences generated from the enrichment bioreactor. In the absence of any related sequences in the database (>92% similarity), the probes were designed against the 16 S sequence of ‘*Ca*. A. nitratireducens’ and care should be taken in their application beyond well characterised systems. Given there are no cultured representatives available for probe validation, three FISH probes were designed to target different sites on the ‘*Ca*. A. nitratireducens’ 16 S rRNA to give higher confidence in their specificity. These included the SYMB-1018 (5ʹ - CCG AAG CCC AGC AAA CTC T − 3ʹ), SYMB-624 (5ʹ- TTC GCA AGC ACT CCC GCA – 3ʹ) and SYMB-186 (5ʹ - TCC TCC CGT CCC CAT GC – 3ʹ) probes. Unlabelled helper probes^78^ were designed to target the flanking regions of each probe site to increase accessibility to the target site for optimal fluorescent signal (SYMB-1018: H1, 5ʹ - ATT TCT AGA GCG GTC AGG GGA TGT − 3ʹ; H2, 5ʹ - CAC CTG TCT CCC TGT CTG GA − 3ʹ; SYMB-624: H1, 5ʹ - GTT AAG CTG CGG GTT TTC ACT CAC − 3ʹ; H2, 5ʹ - CTG CCC TCA AGC CCA ACA GT − 3ʹ; SYMB-186: H1, 5ʹ - GGC CGT GAG CAT ATC CGG TAT TAG C − 3ʹ; H2a, 5ʹ - GAC CCA TCC CGA AGC AGT AAA CCT T − 3ʹ; H2b, 5ʹ - GAC CCA TCC CGA AGT AGC AAC CCT T − 3ʹ). Helper probes were applied in equimolar amounts with their respective probe. The 5ʹ and 3ʹ ends of the oligonucleotide FISH probes were labelled with the Cy3 fluorophore and were synthesized by Integrated DNA Technologies, Singapore. A higher signal was achieved for these FISH probes with lysozyme pre-treatment of the biomass (0.5 mg ml^−1^ in 0.05 M EDTA, 0.1 M Tris-HCl, pH 8) for 30 min at room temperature. The Non-EUB nonsense probe was used as a negative hybridization control^79^. DAPI (1 ng/µl) staining of cells was performed for 15 min in the dark. The labelled biomass was visualized with a Stellaris5 white light laser confocal microscope (Leica, Germany).

### Reporting summary

Further information on research design is available in the Nature Research Reporting Summary linked to this article.

## Supplementary information

Supplementary information

Description of Additional Supplementary Files

Supplementary data 1

Supplementary data 2

Reporting Summary

## Data Availability

Sequencing data are archived in NCBI database under Project number PRJNA802347. All draft genome nucleotide sequences have been submitted to NCBI under accession numbers SAMN25643198 to SAMN25643256. The mass spectrometry proteomics data have been deposited to the ProteomeXchange Consortium via the PRIDE partner repository with the dataset identifier PXD031366. Source data are provided with this paper.

## Source data

Source data file
