# Supplementary material for: Anaerobic oxidation of propane coupled to nitrate reduction by a lineage within the class Symbiobacteriia
Source: Nat Commun. 2022 Oct 17;13:6115. doi: 10.1038/s41467-022-33872-y (PMC9576796; doi:10.1038/s41467-022-33872-y)
Supplement: Supplementary file 1 — Supplementary information [file 41467_2022_33872_MOESM1_ESM.docx]

**Anaerobic oxidation of propane coupled to nitrate reduction by a lineage within the class *Symbiobacteriia***

Mengxiong Wu^1#^, Jie Li^1#^, Andy O Leu^2^, Dirk V Erler^3^, Terra Stark^4^, Gene W. Tyson^2^, Zhiguo Yuan^1^, Simon J. McIlroy^2*^, Jianhua Guo^1*^

^1^Australian Centre for Water and Environmental Biotechnology, Faculty of Engineering, Architecture and Information Technology, The University of Queensland, St Lucia, Queensland, Australia

^2^Centre for Microbiome Research, School of Biomedical Sciences, Queensland University of Technology (QUT), Translational Research Institute, Woolloongabba, Queensland, Australia

^3^Centre for Coastal Biogeochemistry Research, Faculty of Science and Engineering, Southern Cross University, Lismore, New South Wales, Australia

^4^Metabolomics Australia (Queensland Node), Australian Institute for Bioengineering and Nanotechnology, The University of Queensland, St Lucia, QLD 4072, Australia

^#^These authors contributed equally: Mengxiong Wu, Jie Li.

^*^Corresponding author email: [simon.mcilroy@qut.edu.au](mailto:simon.mcilroy@qut.edu.au); jianhua.guo@uq.edu.au.

**1. Supplementary Tables**

**Supplementary Table 1 | Quantification of anaerobic propane oxidation and nitrate reduction by enrichment culture in triplicate batch tests.**

| Nitrogen and electron balance (mmol/L) | C_3_H_8_ oxidized | NO_3_^-^  reduced | NH_4_^+^ generated | N_2_-N generated | Electrons generated by C_3_H_8_ oxidation | Electrons required for NO_3_^-^  reduction^*^ | Nitrogen balance^†^ | Electron balance^‡^ |
| --- | --- | --- | --- | --- | --- | --- | --- | --- |
| 1.84 L culture | -0.30 | -0.82 | 0.45 | 0.39 | 6.07 | 5.49 | 0.99 | 1.11 |
| 0.5 L culture | -0.29 | -0.97 | 0.31 | 0.56 | 5.80 | 5.28 | 1.11 | 1.10 |
| 0.5 L culture | -0.31 | -0.91 | 0.35 | 0.56 | 6.16 | 5.60 | 1.00 | 1.10 |

^*^ Electrons required for NO_3_^-^  reduction = NH_4_^+^ generated$\times$8 + N_2_-N generated$\times$5.

^†^ Nitrogen balance = (NO_3_^-^  reduced)/ (NH_4_^+^ generated + N_2_-N generated ).

^‡^ Electrons generated from propane oxidation divided by electrons required for NH_4_^+^ and N_2_-N production; theoretically higher than 1.0, due to minor fraction of carbon assimilated into biomass cells.

**Supplementary Table 2 |** Microbial community structure of the enrichment culture on Day 1040 analyzed by metagenomic sequencing (≥ 1% relative abundance are shown). Abundance of each genome was calculated using CoverM 0.6.1 (<https://github.com/wwood/CoverM>) with only quality primary mappings.

| **Relative abundance**  **(%)** | **GTDB Classification** |
| --- | --- |
| 22.84 | d__Bacteria;p__Firmicutes_E;c__Symbiobacteriia;o__;f__;g__;s__ |
| 18.05 | d__Bacteria;p__Verrucomicrobiota;c__Verrucomicrobiae;o__Opitutales;f__Opitutaceae;g__Didemnitutus;s__ |
| 7.03 | d__Bacteria;p__Patescibacteria;c__Microgenomatia;o__GWA2-447;f__UBA8517;g__UBA8517;s__ |
| 4.50 | d__Bacteria;p__Bacteroidota;c__Ignavibacteria;o__Ignavibacteriales;f__Melioribacteraceae;g__DSXH01;s__ |
| 4.21 | d__Bacteria;p__Gemmatimonadota;c__Gemmatimonadetes;o__Gemmatimonadales;f__Gemmatimonadaceae;g__SCN-70-22;s__ |
| 3.47 | d__Bacteria;p__Chloroflexota;c__Ellin6529;o__QHBO01;f__QHBO01;g__;s__ |
| 2.81 | d__Bacteria;p__Chloroflexota;c__Anaerolineae;o__Promineofilales;f__Promineofilaceae;g__Promineofilum;s__ |
| 2.34 | d__Bacteria;p__Myxococcota;c__Polyangia;o__Polyangiales;f__Polyangiaceae;g__;s__ |
| 2.33 | d__Bacteria;p__Bacteroidota;c__Kapabacteria;o__Kapabacteriales;f__Kapabacteriaceae;g__OLB6;s__OLB6 |
| 1.03 | d__Bacteria;p__Chloroflexota;c__Dehalococcoidia;o__UBA2991;f__UBA2991;g__UCB2;s__UCB2 |

**Supplementary Table 3 | Genes and proteins involved in ‘*Ca.* A. nitratireducens’ energy metabolism.**

| **Gene** | **Feature of gene product** | **Locus Tag** | **Transcriptome**  **(TPM)** | | **Proteome**  **(Unique peptides)** |  |
| --- | --- | --- | --- | --- | --- | --- |
|  |  |  | **Stage 1** | **Stage 2** |  |  |
| *ATPF0A* | F-type H^+^-transporting ATPase subunit a | 01160 | 701.9 | 462.1 |  | |
| *ATPF0C* | F-type H^+^-transporting ATPase subunit c | 01161 | 3330.2 | 1246.9 | 5 | |
| *ATPF0B* | F-type H^+^-transporting ATPase subunit b | 01162 | 994.8 | 521.1 | 4 | |
| *ATPF1D* | F-type H^+^-transporting ATPase subunit delta | 01163 | 872.6 | 625.9 | 6 | |
| *ATPF1A* | F-type H^+^/Na^+^-transporting ATPase subunit alpha | 01164 | 930.9 | 479.8 | 9 | |
| *ATPF1G* | F-type H^+^-transporting ATPase subunit gamma | 01165 | 1012.9 | 468.0 | 6 | |
| *ATPF1B* | F-type H^+^/Na^+^-transporting ATPase subunit beta | 01166 | 1256.1 | 582.7 | 14 | |
| *ATPF1E* | F-type H+-transporting ATPase subunit epsilon | 01167 | 948.3 | 453.9 | 2 | |
| *nuoN* | NADH-quinone oxidoreductase subunit N | 00540 | 289.5 | 124.5 | 3 | |
| *nuoL* | NADH-quinone oxidoreductase subunit L | 00541 | 210.3 | 99.0 | 2 | |
| *nuoK* | NADH-quinone oxidoreductase subunit K | 00542 | 259.7 | 117.4 |  | |
| *nuoJ* | NADH-quinone oxidoreductase subunit J | 00543 | 315.5 | 141.3 |  | |
| *nuoI* | NADH-quinone oxidoreductase subunit I | 00544 | 336.8 | 149.0 | 3 | |
| *nuoH* | NADH-quinone oxidoreductase subunit H | 00545 | 159.7 | 71.5 | 4 | |
| *nuoG* | NADH-quinone oxidoreductase subunit G | 00547 | 199.0 | 105.3 | 10 | |
| *nuoF* | NADH-quinone oxidoreductase subunit F | 00548 | 300.5 | 165.7 | 10 | |
| *nuoE* | NADH-quinone oxidoreductase subunit E | 00549 | 360.6 | 149.9 | 2 | |
| *nuoD* | NADH-quinone oxidoreductase subunit D | 00550 | 287.8 | 129.4 | 9 | |
| *nuoC* | NADH-quinone oxidoreductase subunit C | 00551 | 295.9 | 142.0 | 7 | |
| *nuoB* | NADH-quinone oxidoreductase subunit B | 00552 | 216.3 | 114.9 | 2 | |
| *nuoA* | NADH-quinone oxidoreductase subunit A | 00553 | 281.6 | 132.3 |  | |
| *nuoA* | NADH-quinone oxidoreductase subunit A | 01966 | 371.9 | 173.4 |  | |
| *nuoB* | NADH-quinone oxidoreductase subunit B | 01967 | 236.3 | 113.0 | 1 | |
| *nuoC* | NADH-quinone oxidoreductase subunit C | 01968 | 352.6 | 181.0 | 3 | |
| *nuoD* | NADH-quinone oxidoreductase subunit D | 01969 | 201.1 | 117.9 | 5 | |
| *nuoH* | NADH-quinone oxidoreductase subunit H | 01970 | 152.4 | 105.4 |  | |
| *nuoI* | NADH-quinone oxidoreductase subunit I | 01971 | 247.1 | 192.5 | 1 | |
| *nuoJ* | NADH-quinone oxidoreductase subunit J | 01972 | 150.5 | 103.3 |  | |
| *nuoK* | NADH-quinone oxidoreductase subunit K | 01973 | 190.2 | 111.0 |  | |
| *nuoL* | NADH-quinone oxidoreductase subunit L | 01974 | 221.1 | 157.6 | 2 | |
| *nuoM* | NADH-quinone oxidoreductase subunit M | 01975 | 480.5 | 209.8 | 4 | |
| *nuoN* | NADH-quinone oxidoreductase subunit N | 01976 | 449.1 | 188.4 | 2 | |

**Supplementary Table 4 | Genes encoding enzymes related to nitrate/nitrite reduction in ‘*Ca.* A. nitratireducens*’.***

| **Gene** | **Feature of gene product** | **Locus Tag** | **Transcriptome**  **(TPM)** | | **Proteome**  **(Unique peptides)** |
| --- | --- | --- | --- | --- | --- |
|  |  |  | **Stage 1** | **Stage 2** |  |
| *napB* | Periplasmic nitrate reductase electron transfer subunit | 00811 | 547.4 | 0.0 | 5 |
| *napA* | Periplasmic nitrate reductase catalytic subunit | 00812 | 340.6 | 8.3 | 34 |
| *nrfA1* | nitrite reductase (cytochrome c-552) | 00493 | 0.0 | 4.0 |  |
| *nrfH1* | cytochrome c nitrite reductase small subunit | 00494 | 0.0 | 0.0 |  |
| *nrfH2* | cytochrome c nitrite reductase small subunit | 01415 | 290.0 | 541.4 |  |
| *nrfA2* | nitrite reductase (cytochrome c-552) | 01416 | 301.9 | 514.1 | 5 |
| *nrfA3* | Putative nitrite reductase (cytochrome c-552) | 01839 | 558.6 | 1945.5 | 4 |
| *nrfH3* | cytochrome c nitrite reductase small subunit | 01840 | 398.8 | 1674.2 |  |
| *norB1* | nitric oxide reductase subunit B | 00401 | 0.0 | 0.0 |  |
| *norB2* | nitric oxide reductase subunit B | 01691 | 1000.6 | 316.9 | 8 |
| *nosD* | nitrous oxidase accessory protein | 02008 | 156.8 | 244.7 |  |
| *nosZ* | nitrous-oxide reductase | 02010 | 611.2 | 1515.9 | 24 |
| *narB1* | Assimilatory nitrate reductase | 00583 | 0.0 | 0.0 |  |
| *narB2* | Assimilatory nitrate reductase | 00584 | 89.8 | 171.5 |  |
| *narK* | nitrate/nitrite transporter | 01241 | 96.2 | 135.0 | 1 |
| *nirC* | nitrite transporter | 00655 | 10.8 | 0.0 |  |
| *nirD* | nitrite reductase (NADH) small subunit | 00656 | 9.6 | 1.3 |  |
| *nirB* | nitrite reductase (NADH) large subunit | 00657 | 0.0 | 0.0 |  |

**Supplementary Table 5 | Operational conditions for the batch test incubations.**

| **Batch test** | **System** | **Biomass/medium** | **Initial NO_3_^-^  concentration (mg/L)** | **Initial added propane (mol)** |
| --- | --- | --- | --- | --- |
| **Stoichiometry determination** | 2.3 L parent reactor | 1.84 L biomass | ~12 | ~29 |
|  | 650 mL reactor | 500 mL biomass | ~14 | ~2.30-2.35 |
| **Abiotic control** | 650 mL reactor | 500 ml sterile medium | ~20 | ~2.30-2.35 |
| **No propane control** | 650 mL reactor | 250 mL biomass | ~15 | × |

**Supplementary Table 6 | Accession numbers of catalytic subunits of alkylsuccinate and benzylsuccinate synthase (AssA and BssA) included in the phylogenetic studies of AssA in ‘*Ca.* A. nitratireducens’.**

| **AssA/BssA (NCBI accession)** | | | | |
| --- | --- | --- | --- | --- |
| CAO03074.1 | ADJ51097.1 | WP012610862.1 | AHI85732.1 |  |
| KUK55592.1 | KUK63464.1 | AMR94128.1 | KFI38250.1 |  |
| KFZ44314.1 | CBK27727.1 | WP_062284143.1 | TWJ18651.1 |  |
| SIR65702.1 | SEF59519.1 | CAA05052.1 | AAK50372.1 |  |
| CAG4882135.1 | WP_006524672.1 | VTR63745.1 | QTQ34323.1 |  |
| AIB50973.1 | AIB50976.1 | HBY57497.1 | SPD74684.1 |  |

**Supplementary Table 7 | Optimized multiple reaction monitoring transitions and retention times for selected compounds analysed by GC-MS/MS.**

| Compound | Retention time (min) | Transient 1 (m/z) | Collision Energy (V) | Transient 2 (m/z) | Collision Energy (V) | Transient 3 (m/z) | Collision Energy (V) |
| --- | --- | --- | --- | --- | --- | --- | --- |
| *Iso*-propyl succinate | 10.832 | 289.0>147.2 | 10 | 217.0>55.1 | 10 | 217.0>73.1 | 20 |
| Propyl succinate | 11.078 | 289.0>147.1 | 10 | 289.0>73.1 | 35 | 262.0>172.1 | 5 |

**2. Supplementary Figures**


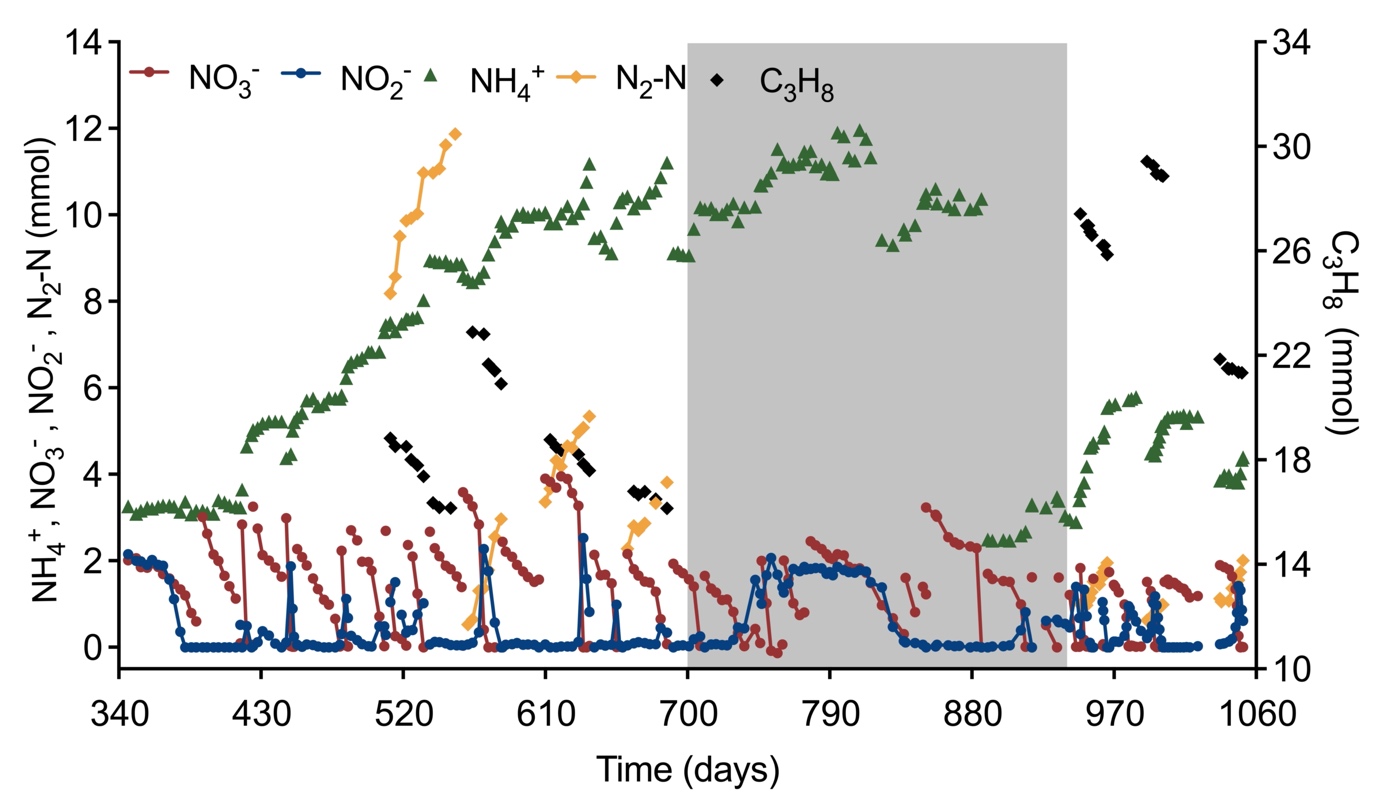


**Supplementary Fig. 1 | Bioreactor long-term performance**. Simultaneous nitrate and propane consumption with transitory formation of nitrite, and production of dinitrogen gas and ammonium. Grey area indicates a lack of data for propane due to a technical issue with the gas chromatograph. Source data are provided as a Source Data file.


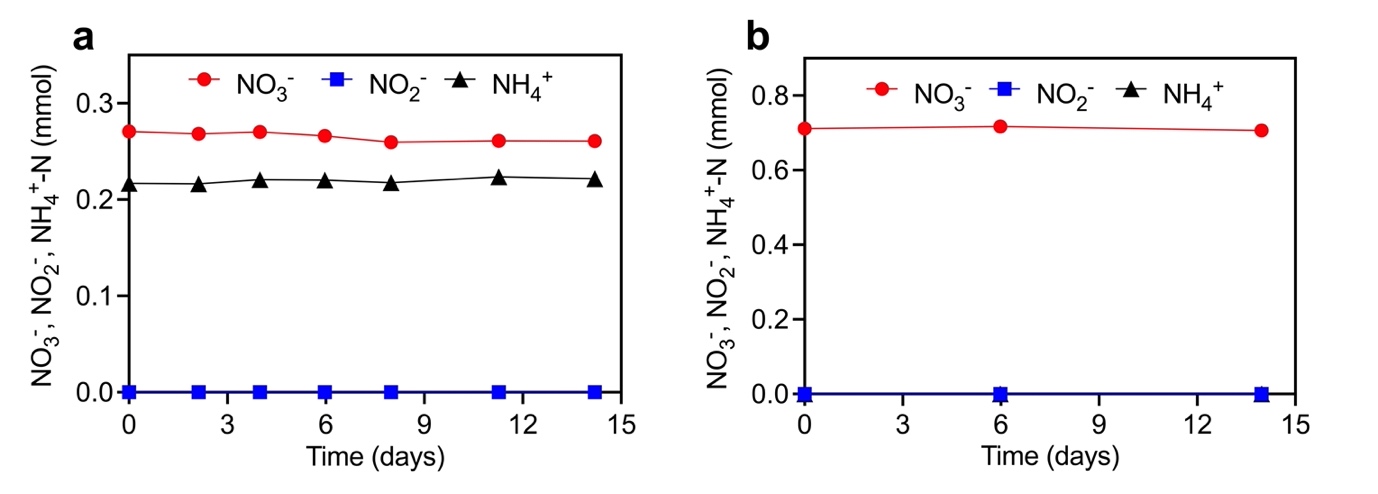


**Supplementary Fig. 2 | Profiles of nitrate, nitrite and ammonium in the control batch incubations.** **a,** no nitrate consumption or ammonium production in the control incubation without propane addition. **b,** no nitrate consumption or ammonium production in the abiotic control incubation without biomass.


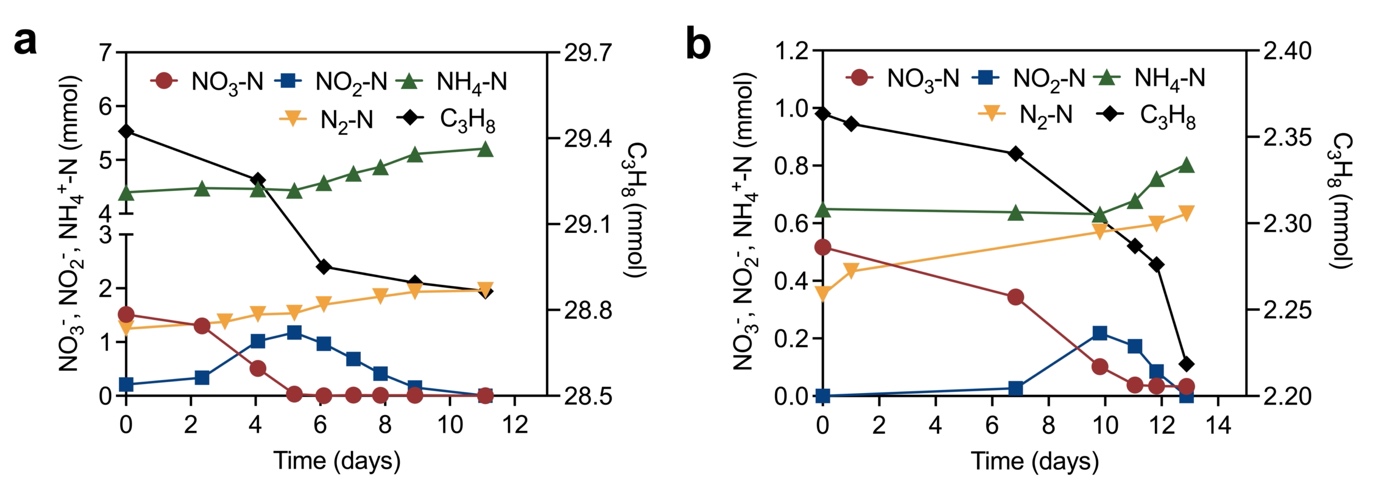


**Supplementary Fig. 3 | Profiles of propane, nitrate, nitrite, ammonium and dinitrogen gas in the batch tests for stoichiometric determination**. **a,** Test started on Day 990 for 1.84 L enrichment culture in the parent reactor. **b,** Test started on Day 1,000 for 0.5 L enrichment culture in the batch incubation. Source data are provided as a Source Data file.


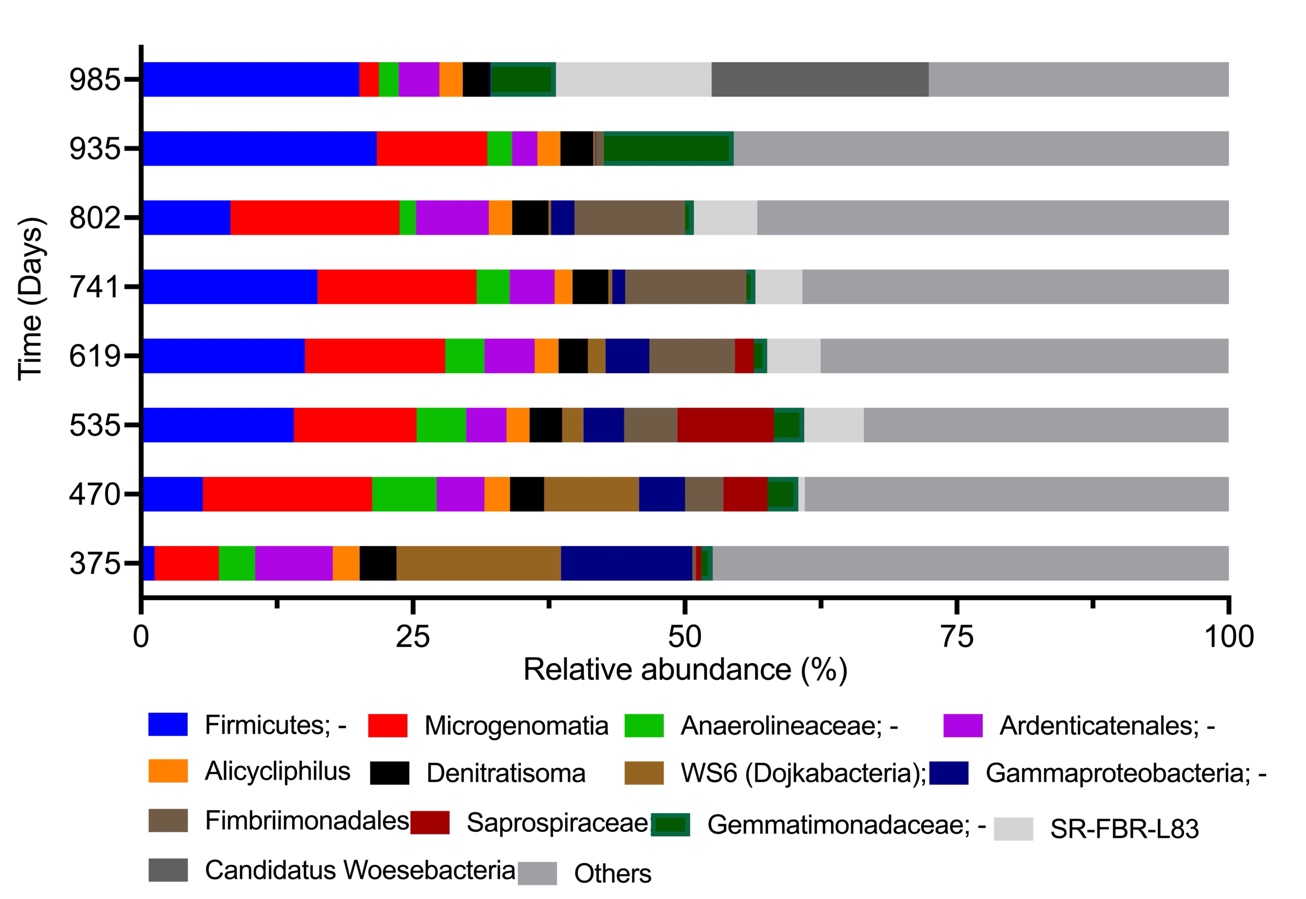


**Supplementary Fig. 4 | 16S rRNA abundance profile of the microbial community during long-term bioreactor operation.** Genera that account for ≥ 2% in at least one sample are shown, while genera with an abundance of less than 2% in all samples are grouped into ‘Others’.


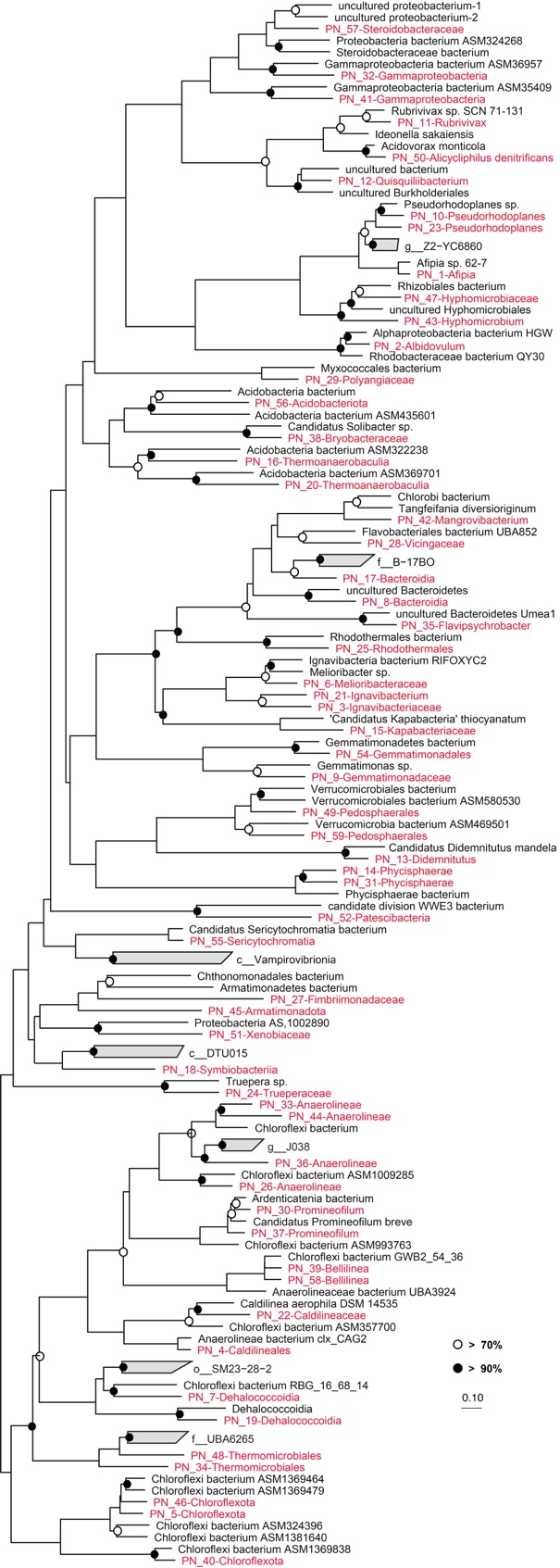


**Supplementary Fig. 5 | Phylogenetic placement of the 59 bacterial population genomes from this study.** A maximum-likelihood tree was constructed with the 59 bacterial population genomes and bacterial reference genomes in the Genome Taxonomy Database using a concatenated set of 120 bacterial-specific marker genes. Bootstrap values were determined by non-parametric bootstrapping of 100 replicates. The population genomes from this study are highlighted in red. Black and white dots represent >90% and >70% bootstrap values, respectively. The scale bar indicates the number of amino acid substitutions per site.

**
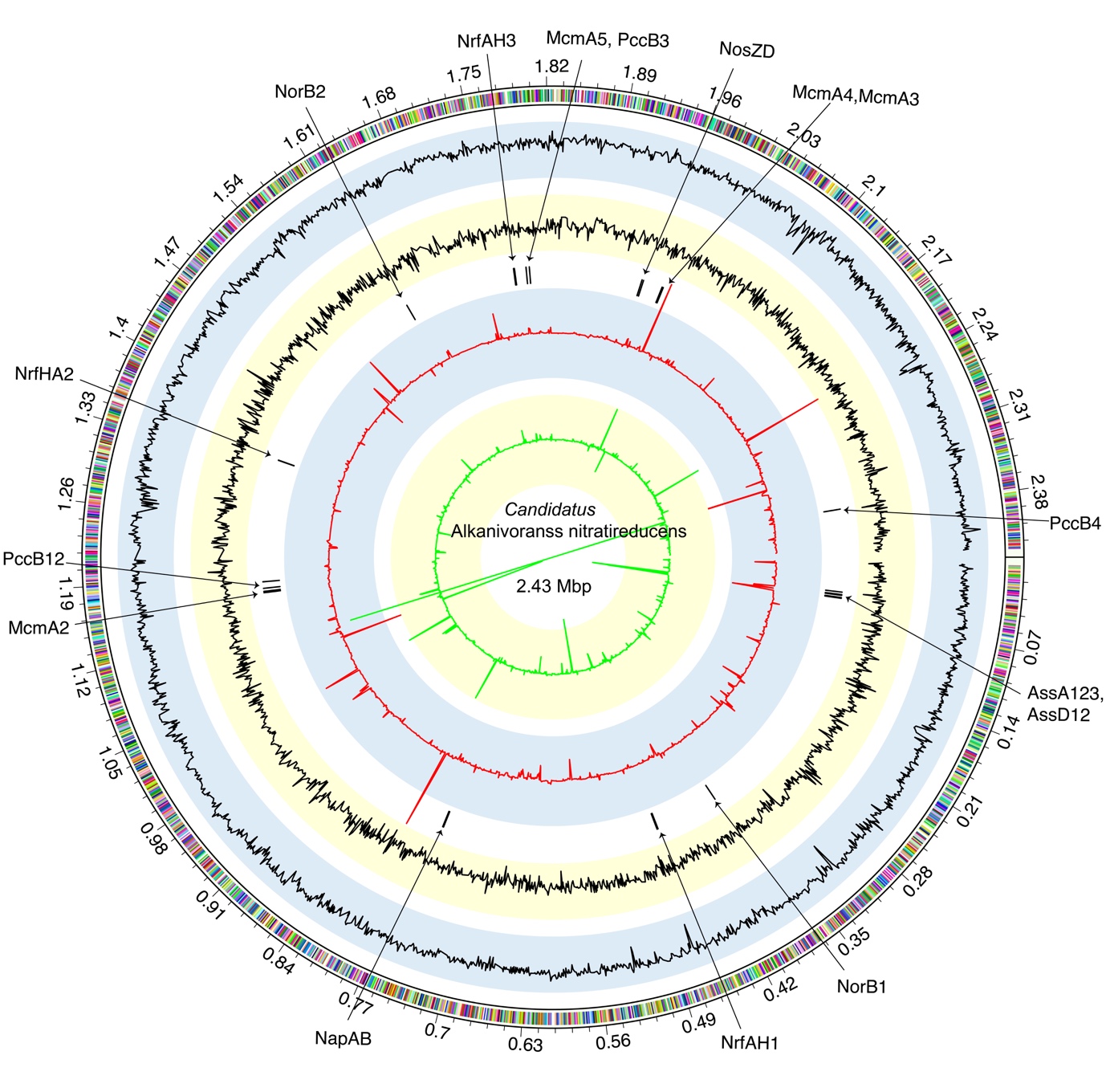
**

**Supplementary Fig. 6 | Features of the circular genome ‘*Ca*. A. nitratireducens’.** From outside to inside: encoded genes (ring 1 with coordinates), GC content (ring 2), GC skew (ring 3), genes of enzymes related to nitrogen and propane metabolism (ring 4), RNA expression levels of genes in Stage 1 (ring 5) and Stage 2 (ring 6). RNA expression of genes encoded on ‘+’ strand was plotted toward outside while the expression of genes encoded on ‘-’strand was toward inside. Ass, Alkylsuccinate synthase; Mcm, Methylmalonyl-CoA mutase; Pcc, Propionyl-CoA carboxylase; Nap, Nitrate reductase; Nrf, Cytochrome *c* 552 nitrite reductase; Nor, Nitric oxide reductase; Nos, Nitrous oxide reductase.


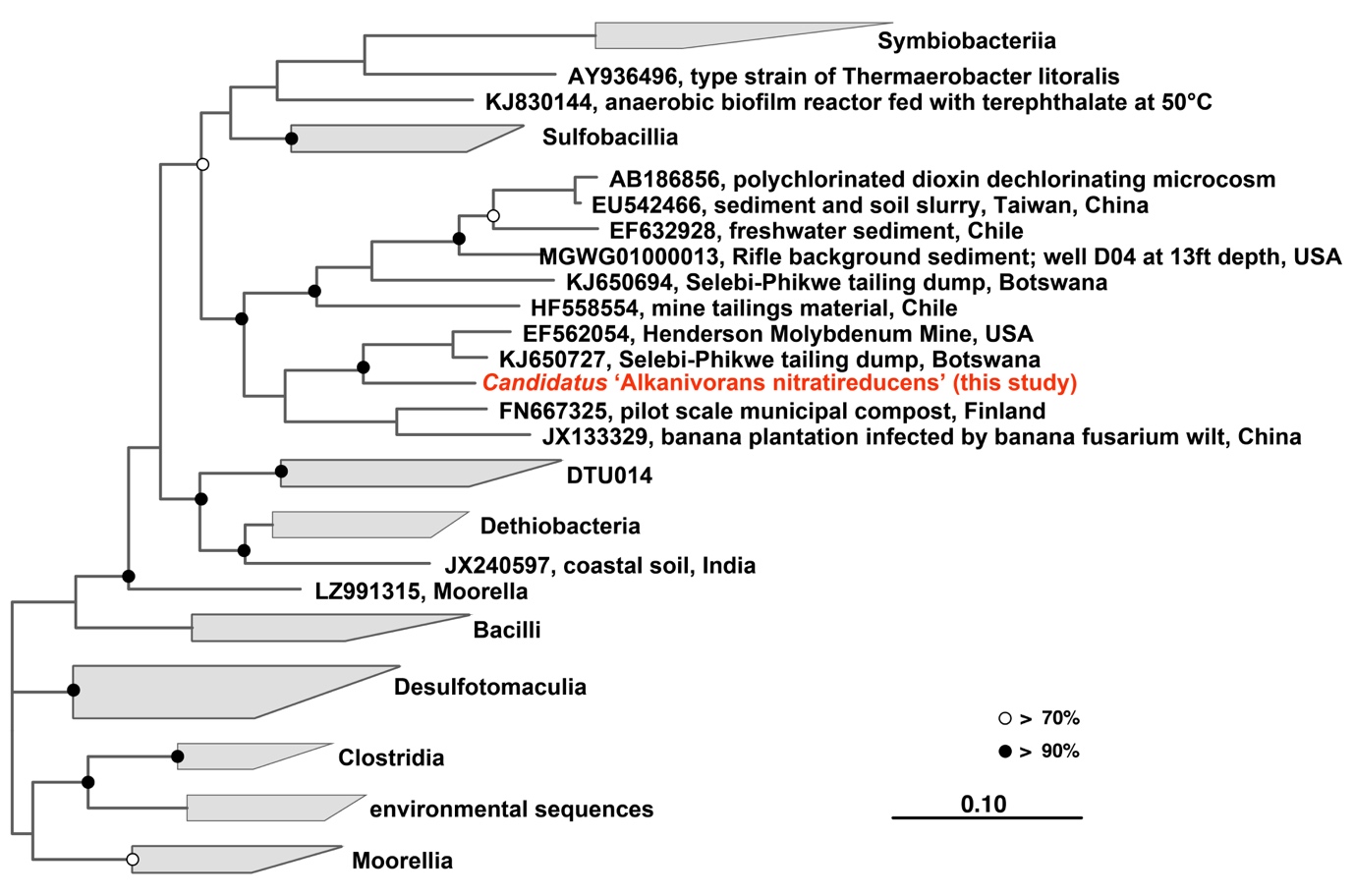


**Supplementary Fig. 7 | Phylogenetic affiliation of the dominant ‘*Ca*. A. nitratireducens’ (16S rRNA tree).** 16S rRNA sequence from this study are highlighted in red. Black and white dots represent >90% and >70% bootstrap values, respectively. The scale bars indicate nucleotide substitutions per site.


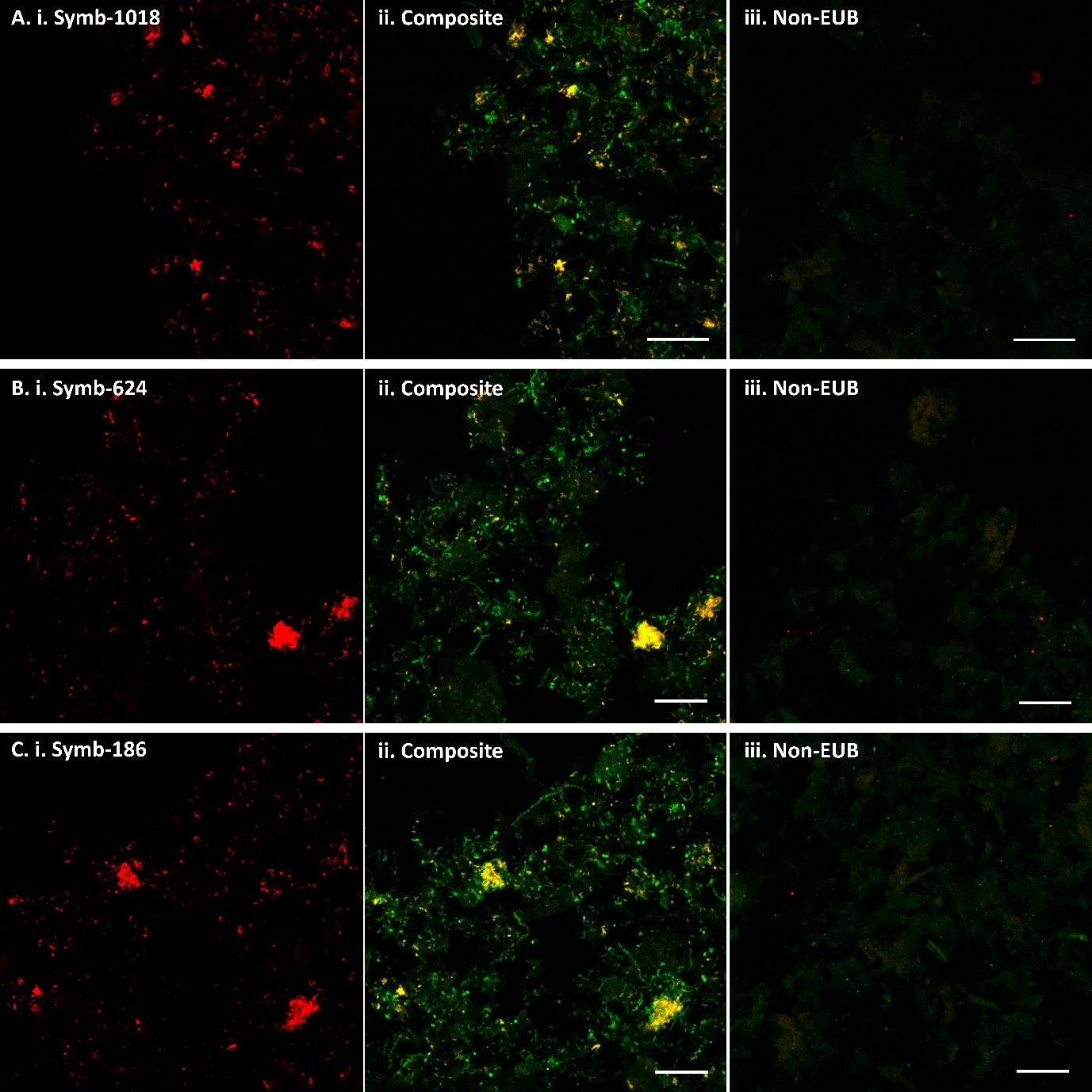


**Supplementary Fig. 8 |** Fluorescence *in situ* hybridisation (FISH) micrographs of the bioreactor biomass. **A.-C. i.** Probes targeting ‘*Ca.* A. nitratireducens’ (Cy3 label, red; probes indicated on image); **ii.** Same field of view composite image including EUBmix probe set^2,3^ (Fluorescein isothiocyanate label, green; All bacteria). ‘*Ca.* A. nitratireducens’ cells appear yellow (red + green) and other bacterial cells appear green. **iii.** Non-EUB (Cy3 label, red) negative control with ‘red’ and ‘green’ channels taken with the same settings. The scale bar indicates 20 μm. The representative images were selected based on the visual assessment of >3 separate hybridisation experiments.


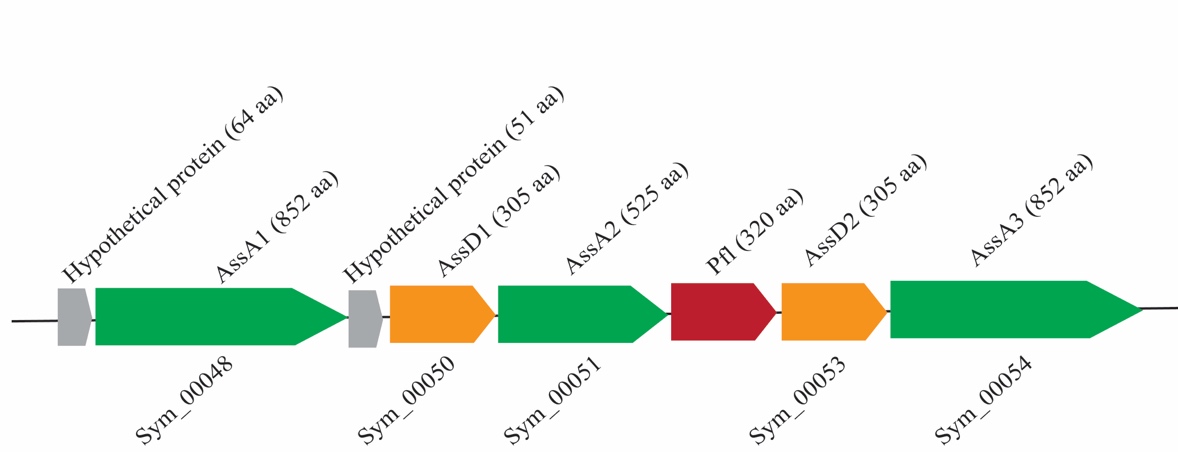


**Supplementary Fig. 9 | Alkylsuccinate synthase gene loci in ‘*Ca*. A. nitratireducens’.** Three catalytic subunits (AssA123) which are highly similar (amino acid sequences of AssA2 and AssA3 showed 90.7% and 89.3% similarities with AssA1, respectively) and two radical activating subunits of alkylsuccinate synthase (AssD12) which have identical amino acid sequences, were identified in the Ass protein complexes. No putative beta (AssB) or gamma subunits (AssC) were found in the genome.


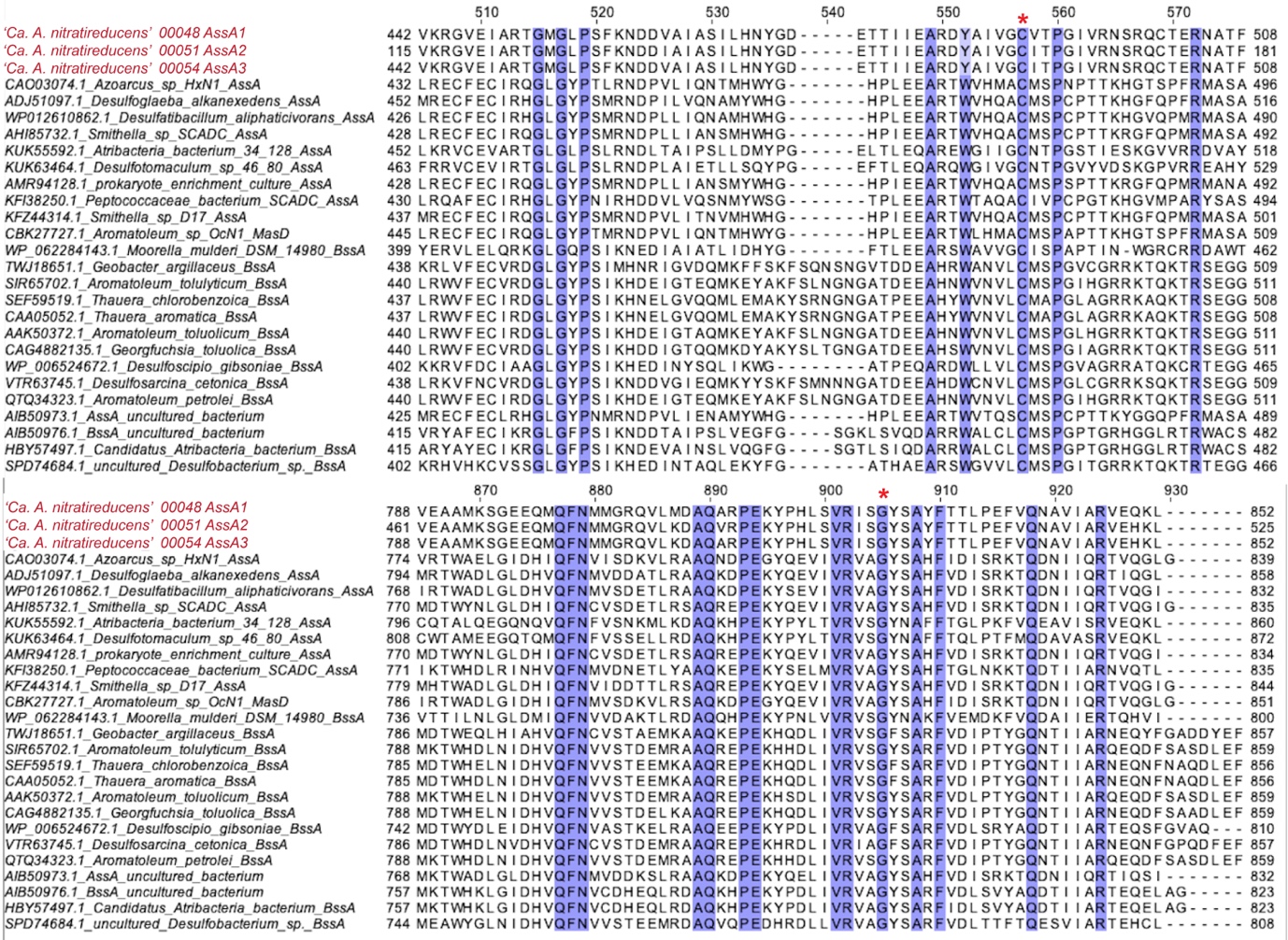


**Supplementary Fig. 10 | Partial sequence alignment of alkylsuccinate and benzylsuccinate synthase alpha subunits (AssA and BssA) from ‘*Ca*. A. nitratireducens’ and selected reference microorganisms.** Three AssA in ‘*Ca*. A. nitratireducens’ (red) are aligned to AssA and BssA amino acid sequences in the NCBI database (sequence name start with the NCBI identifiers, followed by the microorganism and protein names). Highly conserved positions in AssA and BssA are highlighted in blue. Key conserved amino acid residues as identified in the study of Heider and colleagues^4^ are marked with “*”.


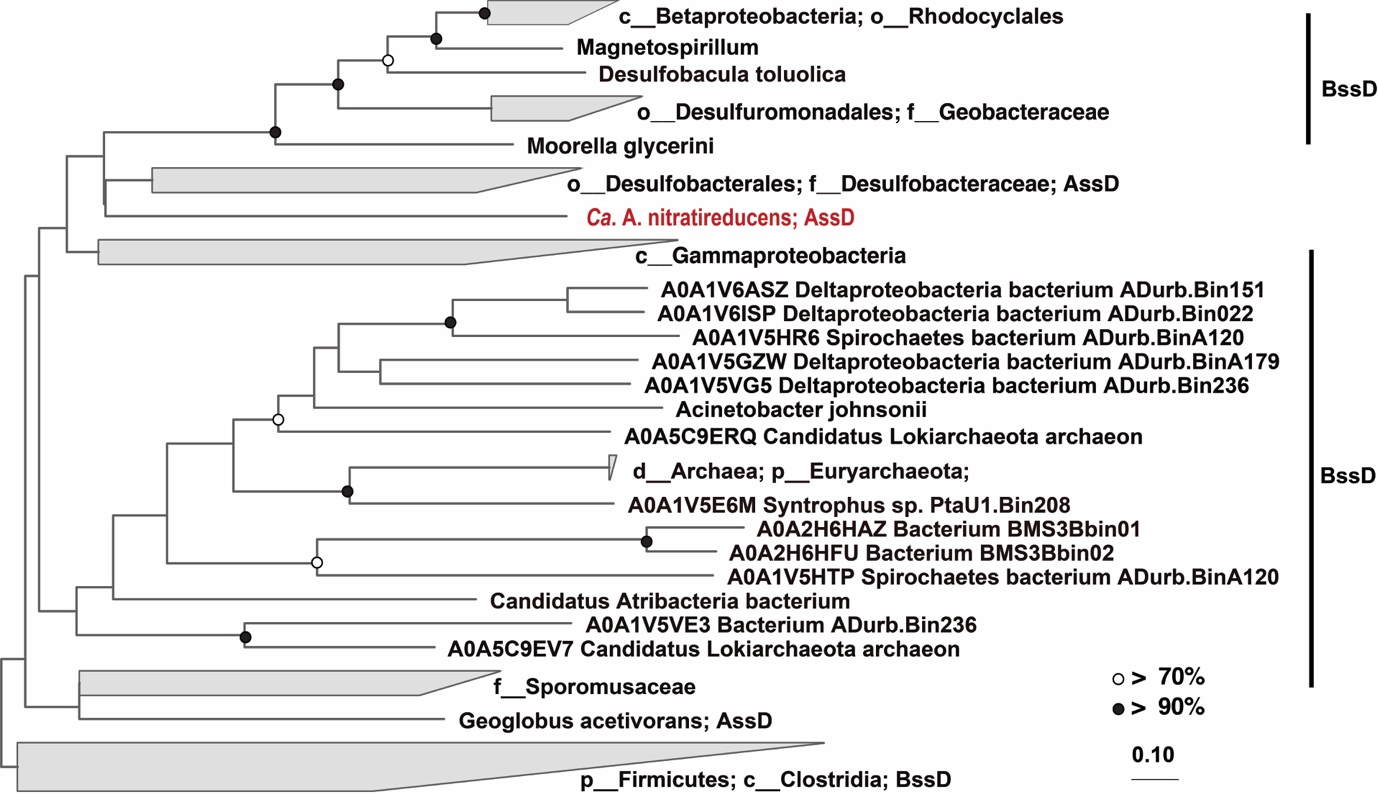


**Supplementary Fig. 11 |** **Phylogenetic affiliation of alkylsuccinate synthase activating enzymes (AssD) in** *‘****Ca.* A. nitratireducens*’* genome.** AssD retrieved from *‘Ca.* A. nitratireducens*’* MAG was highlighted in red. Black and white dots represent >90% and >70% bootstrap values, respectively. The scale bars indicate amino acid substitution per site.

*
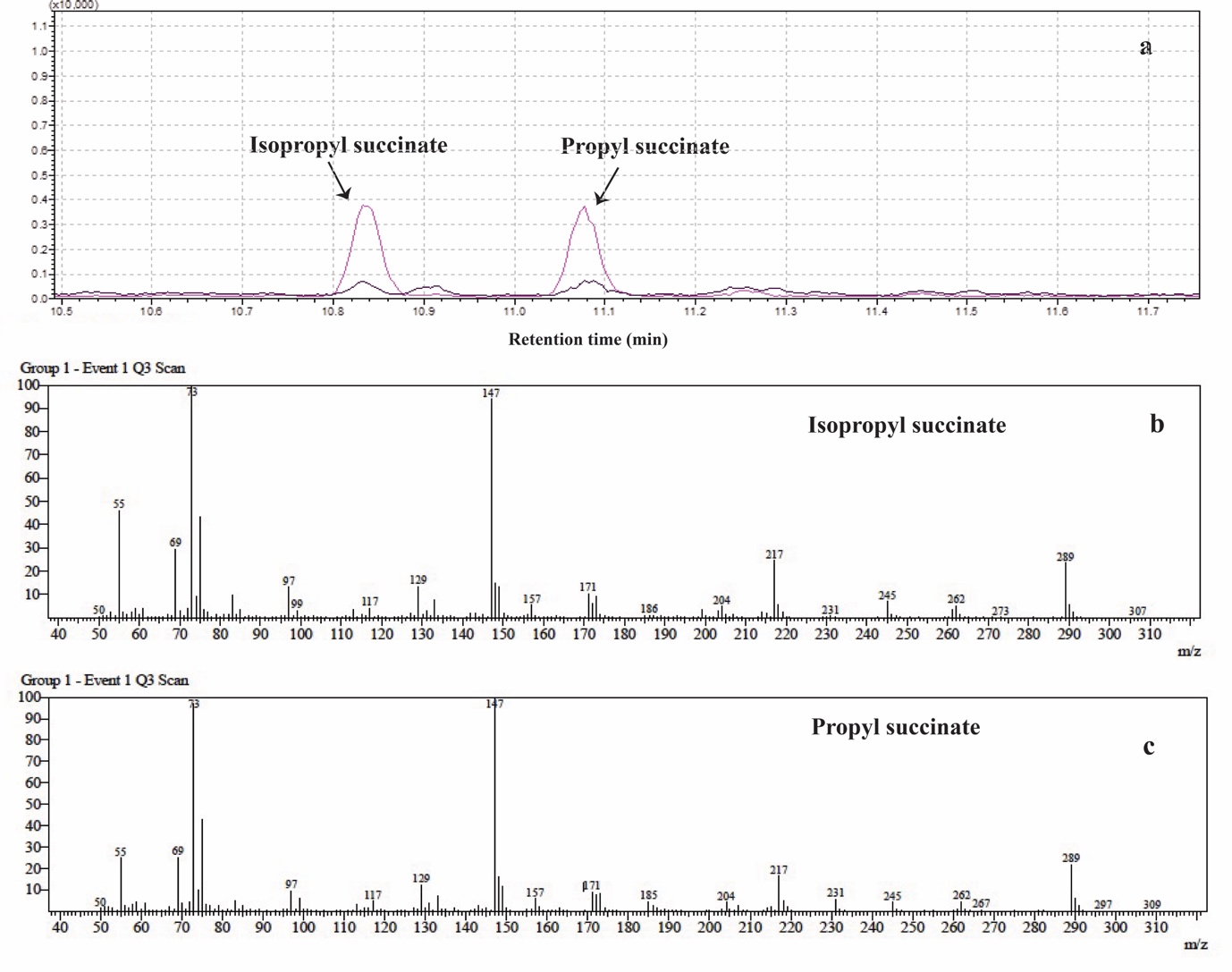
*

**Supplementary Fig. 12 | a.** Total ion chromatograms of the culture extracts (black line) and mixed standards of *iso-*propyl and propyl succinate (Pink line). **b, c.** Mass spectrum of *iso*-propyl (b) and propyl (c) succinate standards.


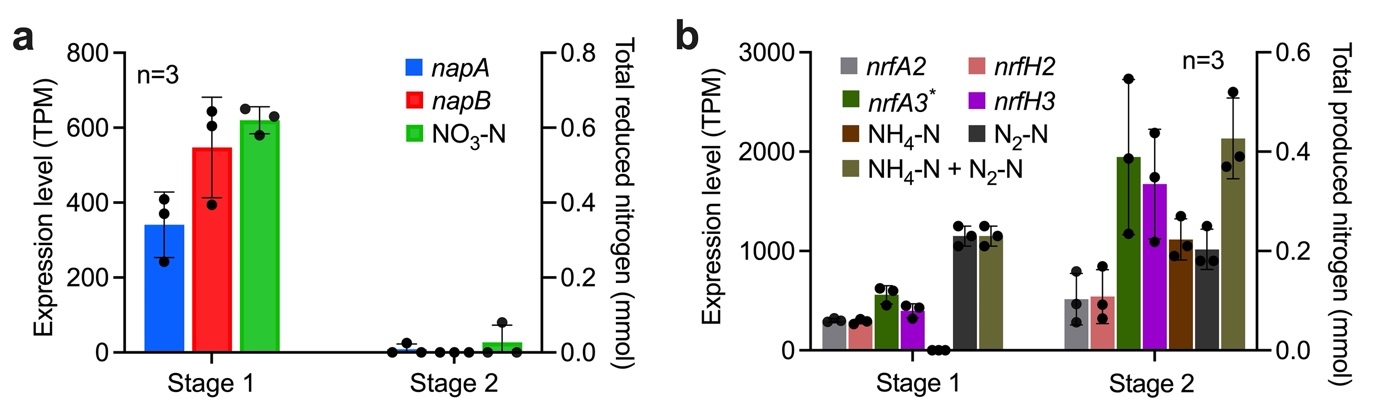


**Supplementary Fig. 13 |** **Expression levels of *napAB* and *nrfAH* (TPM), and total amount of nitrate reduced and dinitrogen gas and ammonium generated in Stage 1 and 2 of triplicate batch tests. a,** High expression of *napAB* in Stage 1, with very low expression in Stage 2, consistent with the total amount of nitrate reduced in each stage. Error bars represent standard deviations. **b,** High expression of *nrfAH* in Stage 1 with generation of dinitrogen gas but not ammonium, and significantly higher expression of *nrfAH* in Stage 2, with accordingly higher amount of dinitrogen gas and ammonium generated. Error bars represent standard deviations. Source data are provided as a Source Data file.

**
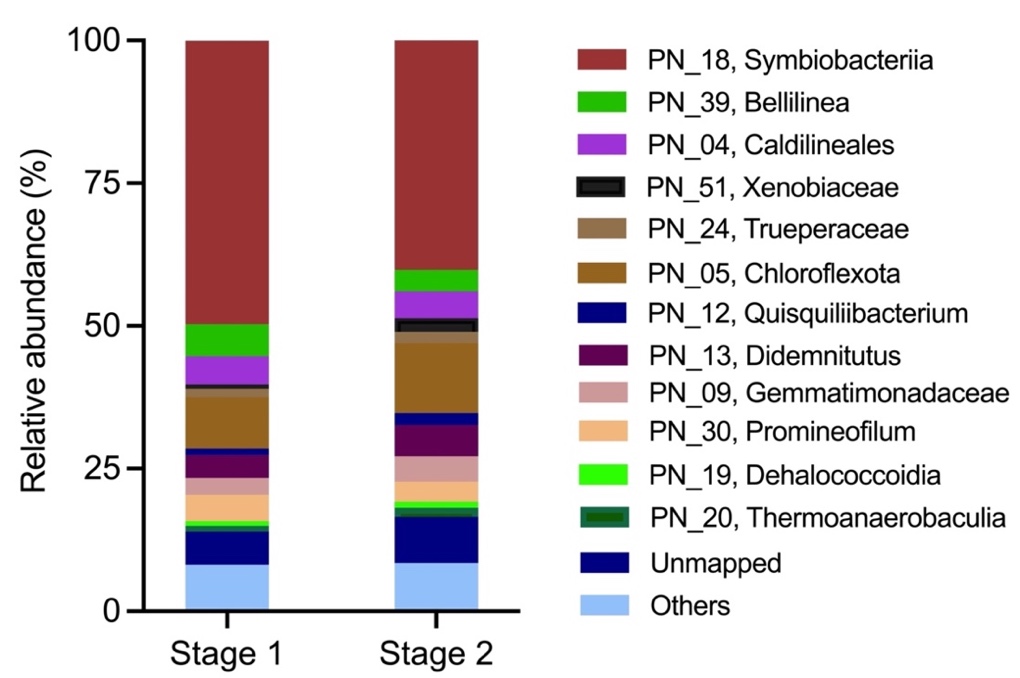
**

**Supplementary Fig. 14 | Relative abundance of the dereplicated genome set during batch tests conducted on Day 1100.** Relative abundance was calculated based on mapping of quality metagenomic reads. Genome sets and unmapped contigs that account for ≥ 1% (average values calculated based on samples from triplicate batch reactors) are shown, while genome sets with an abundance less than 1% are grouped into “Others”.

1. Parks, D. H., Imelfort, M., Skennerton, C. T., Hugenholtz, P. & Tyson, G. W. CheckM: assessing the quality of microbial genomes recovered from isolates, single cells, and metagenomes. *Genome Res.* **25**, 1043-1055 (2015).

2. Amann, R. I. *et al.* Combination of 16S rRNA-targeted oligonucleotide probes with flow cytometry for analyzing mixed microbial populations. *Appl. Environ. Microbiol.* **56**, 1919-1925 (1990).

3. Daims, H., Brühl, A., Amann, R., Schleifer, K. H., Wagner, M. The domain-specific probe EUB338 is insufficient for the detection of all Bacteria: development and evaluation of a more comprehensive probe set. *Sys.Appl.Microbiol.* **22**, 443-444 (1999).

4. Heider, J. *et al.* Structure and Function of Benzylsuccinate Synthase and Related Fumarate-Adding Glycyl Radical Enzymes. *J. Mol. Microbiol. Biotechnol.* **26**, 29–44 (2016)
