# Supplementary material for: Anaerobic oxidation of propane coupled to nitrate reduction by a lineage within the class Symbiobacteriia
Source: Nat Commun. 2022 Oct 17;13:6115. doi: 10.1038/s41467-022-33872-y (PMC9576796; doi:10.1038/s41467-022-33872-y)
Supplement: Supplementary file 2 — Description of Additional Supplementary Files [file 41467_2022_33872_MOESM2_ESM.pdf]

**Description of Additional Supplementary Files:**

**Supplementary Data 1 |** Key features of the metagenome (short-read and long-read sequencing) and metatranscriptome datasets generated for the enrichment culture, and the 59 draft genomes recovered from the metagenomic data (≥70% completeness and ≤10% contamination). Assessments of the genomes were based on the CheckM program.

**Supplementary Data 2 |** Genes encoding enzymes for propane activation and complete oxidation in '*Ca.* A. nitratireducens'.
