# Supplementary material for: Anaerobic oxidation of propane coupled to nitrate reduction by a lineage within the class Symbiobacteriia
Source: Nat Commun. 2022 Oct 17;13:6115. doi: 10.1038/s41467-022-33872-y (PMC9576796; doi:10.1038/s41467-022-33872-y)
Supplement: Supplementary file 5 — Reporting Summary [file 41467_2022_33872_MOESM5_ESM.pdf]

nature portfolio

Corresponding author(s): Jianhua Guo

Last updated by author(s): Sep 30, 2022

Reporting Summary

Nature Portfolio wishes to improve the reproducibility of the work that we publish. This form provides structure for consistency and transparency in reporting. For further information on Nature Portfolio policies, see our Editorial Policies and the Editorial Policy Checklist.

Statistics

For all statistical analyses, confirm that the following items are present in the figure legend, table legend, main text, or Methods section.

n/a	Confirmed	
☐	☒	The exact sample size (*n*) for each experimental group/condition, given as a discrete number and unit of measurement
☐	☒	A statement on whether measurements were taken from distinct samples or whether the same sample was measured repeatedly
☒	☐	The statistical test(s) used AND whether they are one- or two-sided<br>*Only common tests should be described solely by name; describe more complex techniques in the Methods section.*
☒	☐	A description of all covariates tested
☒	☐	A description of any assumptions or corrections, such as tests of normality and adjustment for multiple comparisons
☐	☒	A full description of the statistical parameters including central tendency (e.g. means) or other basic estimates (e.g. regression coefficient) AND variation (e.g. standard deviation) or associated estimates of uncertainty (e.g. confidence intervals)
☒	☐	For null hypothesis testing, the test statistic (e.g. *F*, *t*, *r*) with confidence intervals, effect sizes, degrees of freedom and *P* value noted<br>*Give P values as exact values whenever suitable.*
☒	☐	For Bayesian analysis, information on the choice of priors and Markov chain Monte Carlo settings
☒	☐	For hierarchical and complex designs, identification of the appropriate level for tests and full reporting of outcomes
☒	☐	Estimates of effect sizes (e.g. Cohen's *d*, Pearson's *r*), indicating how they were calculated

Our web collection on statistics for biologists contains articles on many of the points above.

Software and code

Policy information about availability of computer code

Data collection	Chemstation software C.01.10 (Agilent, United States), Thermo Proteome Discoverer (version 2.2.0.388)
Data analysis	Adobe Illustrator 2021, ReadTrim v0.0.1(https://github.com/jlli6t/ReadTrim), FastQC (https://www.bioinformatics.babraham.ac.uk/projects/fastqc/), FastUniq-1.1, cutadapt 2.10, Trimmomatic-0.36, Albacore (https://github.com/Albacore/albacore), Porechop v0.2.4 (https://github.com/rrwick/Porechop), Aviary (https://github.com/rhysnewell/aviary), Bandage v0.90, DASTools 1.1.2, drep-3.2.2, IGV 2.11.1, CheckM v1.1.3, bowtie 2.3.4.3, CoverM 0.6.1, BioSut (https://github.com/jlli6t/BioSut), Prokka 1.14.5, kofamscan 1.3.0, diamond v2.0.11.149, emapper 2.1.5, Prodigal 2.6, HMMER 3.3, FastTree 2.1.11, GenomeTreeTk v0.1.6 (https://github.com/dparks1134/GenomeTreeTk), ARB 6.0.6, cd-hit 4.8.1, SSU-align v0.1.1, muscle 3.8.31, trimAI 1.4.1, SortMeRNA 4.2.0, RNAdir (https://github.com/jlli6t/RNAdir), dirseq v0.4.3 (https://github.com/wwood/dirseq).

For manuscripts utilizing custom algorithms or software that are central to the research but not yet described in published literature, software must be made available to editors and reviewers. We strongly encourage code deposition in a community repository (e.g. GitHub). See the Nature Portfolio guidelines for submitting code & software for further information.

Data

Policy information about availability of data

All manuscripts must include a data availability statement. This statement should provide the following information, where applicable:

- Accession codes, unique identifiers, or web links for publicly available datasets
- A description of any restrictions on data availability
- For clinical datasets or third party data, please ensure that the statement adheres to our policy

Sequencing data are archived in NCBI database under Project number PRJNA802347 (https://www.ncbi.nlm.nih.gov/bioproject/PRJNA802347). All draft genome

nucleotide sequences have been submitted to NCBI under accession numbers SAMN25643198–25643256. The mass spectrometry proteomics data have been deposited to the ProteomeXchange Consortium via the PRIDE partner repository with the dataset identifier PXD031366.

Field-specific reporting

Please select the one below that is the best fit for your research. If you are not sure, read the appropriate sections before making your selection.

☒ Life sciences ☐ Behavioural & social sciences ☐ Ecological, evolutionary & environmental sciences

For a reference copy of the document with all sections, see nature.com/documents/nr-reporting-summary-flat.pdf

Life sciences study design

All studies must disclose on these points even when the disclosure is negative.

Sample size	Sample sizes are based on multiple past experiences with the analytical instruments in this and other laboratories (Haroon, M. F. et al. Nature 500, 567-570 (2013); Cai, C. et al. ISME J. 12, 1929–1939 (2018); Laso-Pérez, R. et al. Nature 539, 396-401 (2016).). The sample sizes which varied for each investigation are detailed in the Methods section.
Data exclusions	No data were excluded from the analysis
Replication	The enrichment culture was obtained by long-term (>1,000 days) bioreactor operation with reproducible performance (n>10).<br>Mass and electron balance tests were conducted with n = 3 independent cultures with different starting concentrations of propane.<br>Isotope experiment was conducted with time series sampling strategy (at least 5 sampling points) on one independent culture.<br>Metabolite analyses were performed with metabolite extracts from n = 3 independent cultures.<br>Metatranscriptomics and metaproteomic analyses were conducted with n = 3 independent cultures at each stage. For metagenomics, the main aim is to obtain genomes from the cultures, therefore, one sample was taken for short-read sequencing and also one sample for long-read sequencing. Community structures at metatranscriptomics and metaproteomic sampling points (as revealed by shallow metagenomics sequencing, n=3 each stage) were consistent with the structure analyzed by the initial metagenome sequencing.<br>The representative FISH image was selected based on the visual assessment of six separate hybridisation experiments. Consistent results were also obtained independently with two additional probes targeting the ‘Ca. A. nitratireducens’ population.<br>The representative images in supplementary Fig. 8 were selected based on the visual assessment of >3 separate hybridisation experiments.<br>All attempts at replication were successful.
Randomization	The experiments were not randomized, since all experiments in this study (mass and electron balance, isotope experiment, meta-omics data analyses) were based on enrichment culture maintained in the lab with replicates.
Blinding	No blinding was performed in this study, because all experiments were conducted with the unique anaerobic enrichment culture carefully maintained in the bioreactor, which made blinding such studies not applicable.

Reporting for specific materials, systems and methods

We require information from authors about some types of materials, experimental systems and methods used in many studies. Here, indicate whether each material, system or method listed is relevant to your study. If you are not sure if a list item applies to your research, read the appropriate section before selecting a response.

Materials & experimental systems

n/a	Involved in the study	
☒	☐	Antibodies
☒	☐	Eukaryotic cell lines
☒	☐	Palaeontology and archaeology
☒	☐	Animals and other organisms
☒	☐	Human research participants
☒	☐	Clinical data
☒	☐	Dual use research of concern

Methods

n/a	Involved in the study	
☒	☐	ChIP-seq
☒	☐	Flow cytometry
☒	☐	MRI-based neuroimaging
